# Feasibility of reintroducing grassland megaherbivores, the greater one-horned rhinoceros, and swamp buffalo within their historic global range

**DOI:** 10.1038/s41598-021-83174-4

**Published:** 2021-02-24

**Authors:** Harshini Y. Jhala, Qamar Qureshi, Yadvendradev V. Jhala, Simon A. Black

**Affiliations:** 1 Durrell Institute of Conservation and Ecology, School of Anthropology and Conservation, University of Kent, Canterbury, CT2 7NZ UK; 2 Wildlife Institute of India, Chandrabani, Dehradun, Uttarakhand 248001 India

**Keywords:** Conservation biology, Ecological modelling, Grassland ecology, Population dynamics, Restoration ecology, Tropical ecology, Conservation biology, Ecological modelling, Grassland ecology, Population dynamics, Restoration ecology

## Abstract

Reintroduction of endangered species is an effective and increasingly important conservation strategy once threats have been addressed. The greater one-horned rhinoceros and swamp buffalo have declined through historic hunting and habitat loss. We identify and evaluate available habitat across their historic range (India, Nepal, and Bhutan) for reintroducing viable populations. We used Species Distribution Models in Maxent to identify potential habitats and evaluated model-identified sites through field visits, interviews of wildlife managers, literature, and population-habitat viability analysis. We prioritize sites based on size, quality, protection, management effectiveness, biotic pressures, and potential of conflict with communities. Our results suggest that populations greater than 50 for rhinoceros and 100 for buffalo were less susceptible to extinction, and could withstand some poaching, especially if supplemented or managed as a metapopulation. We note some reluctance by managers to reintroduce rhinoceros due to high costs associated with subsequent protection. Our analysis subsequently prioritised Corbett and Valmiki, for rhino reintroduction and transboundary complexes of Chitwan-Parsa-Valmiki and Dudhwa-Pilibhit-Shuklaphanta-Bardia for buffalo reintroductions. Establishing new safety-nets and supplementing existing populations of these megaherbivores would ensure their continued survival and harness their beneficial effect on ecosystems and conspecifics like pygmy hog, hispid hare, swamp deer, hog deer, and Bengal florican.

## Introduction

Species are facing an unprecedented extinction crisis due to anthropogenic impacts^1^ with large carnivores and megaherbivores bearing the brunt^2^.These taxa have been extirpated from many of their natural habitats by direct hunting for meat, trophies, crop protection, and retaliatory killing^3,4^. However, large carnivores have been observed to recolonize areas where these threats have been removed, if such areas are connected with source populations^5^. Habitat connectivity for megaherbivores differs to connectivity in carnivores, since carnivores can often pass through degraded habitats, while megaherbivore corridors require forage, water and absence form potential conflict with humans. Megaherbivores share slow life history traits, which make them more vulnerable to threats of habitat loss and poaching^6^. As a consequence, recolonization is rarely observed in megaherbivores other than in elephants^7^, since most source populations are depleted and connecting corridors are degraded or destroyed. Planned reintroductions can be a useful strategy to establish safety-net populations and are often a better option, since natural recolonization is a slow and stochastic process^8^.

Megaherbivore species are largely unaffected by non-human predation, so can sustain high stable densities, allowing them to greatly modify the ecosystems they inhabit^9^. As ecosystem engineers, megaherbivores regulate tall coarse grass through grazing and trampling, which allows the growth of palatable species accessible for consumption by smaller mesoherbivore species^10,11^. Reintroduction and supplementation of megaherbivores would not only create safety-net populations but also restore an important ecological role in currently degraded habitats^12^ and recover the potential of such habitats to sustain historical faunal assemblages.

The Indian subcontinent is home to a diverse range of megaherbivores, including elephant (*Elephus maximus*), gaur (*Bos gaurua*), greater *one-horned* rhinoceros (*Rhinoceros unicornis*) (hereafter ‘rhinoceros’), and wild swamp buffalo *(Bubalus arnee*) (hereafter ‘buffalo’). In recent centuries the populations of rhinoceros and buffalo, both grassland dependent species, have severely declined. Historically, rhinoceros were distributed and abundant across the foothills and floodplains of the Indus (Sindh in Pakistan), Ganges, and the Brahmaputra (up to the Indo-Myanmar border)^13^. Current rhinoceros range is limited and fragmented across India and Nepal as a result of anthropogenic effects^14^. The current global population of rhinoceros is estimated at ~ 3500, of which Kaziranga National Park (NP) in Assam, India (~ 2400 individuals) and Chitwan NP in Nepal (~ 600 individuals) being the only major population strongholds^15^. Similarly, the buffalo was once abundant across the northern and central plains of the subcontinent (from the Indus basin to Brahmaputra floodplains and into south China and Southeast Asia), but is now restricted to small pockets in north-eastern and central India, Nepal, Bhutan, Myanmar, Cambodia and Thailand, with an estimated population of 3000–4000 individuals^16^.

Buffalo and rhinoceros exemplify conservation problems faced by megaherbivores; the rhinoceros more so by being severely persecuted for its horn^17^, whilst the buffalo suffers occasional poaching for bushmeat^16^ and populations of both species have dwindled due to habitat loss. The majority of flood-plain and foot-hill habitat has been converted to agriculture and remaining habitat within Protected Areas (PA) is vulnerable to erosion caused by floods and invasion of exotic plant species like *Mikania micantha, Mimosa invisa* and *Leea crispa*^14^*.* Conflict with people due to economic loss from crop-raiding and occasional human casualties result in negative attitudes in local communities which impedes conservation efforts^18^.

Alongside other criteria, IUCN red listing of species depends on the number and size of populations; with a greater number of large populations endowing higher chances of species survival^19^. Therefore, long-term conservation of megaherbivores depends on establishing viable populations across their global range. This could be achieved through reintroductions into suitable habitats and supplementing existing populations. We use species current occurrence data to model their potential distributions using Maximum Entropy algorithms (Maxent)^20^. These model-identified habitats were evaluated by field surveys, literature review, and discussions with PA managers. We subsequently assessed the feasibility of reintroduction and supplementation based on population habitat viability analysis (PHVA^21^) using parameters of varying levels of poaching, habitat quality, and size. Our study combines information from Species Distribution Models (SDMs), PHVA, management effectiveness, and potential for conflict with humans, in order to prioritize sites for reintroduction and supplementation. We also provide recommendations for enhancing conservation management of extant populations.

## Results

### Species distribution modeling

#### Greater one-horned rhinoceros

**Figure 1:**
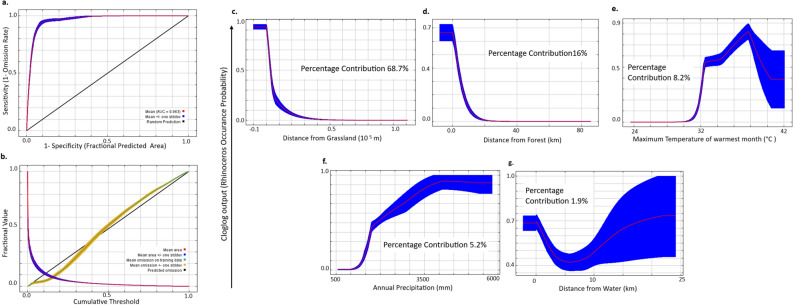
(**a**) Receiver operator curve for assessing model fit, (**b**) Omission/Commission analysis for model accuracy of classifying test data. Species occurrence probability (response curves) obtained from 100 bootstrap runs of the best model explaining the distribution of greater one horned rhinoceros in Maxent to (**c**) distance to grassland, (**d**) distance to forest, (**e**) maximum temperature of warmest month, (**f**) annual precipitation, and (**g**) distance to water.

Based on Area Under the Curve (AUC) of the Receiver Operator Curve (ROC) and on 100 bootstrap runs of omission/commission analysis with 20% test data showed that the rhinoceros model had a good fit [AUC = 0.96 (SE 0.0007)] and predictive ability (Fig. 1a,b). This model was also rated the best by True Skill Statistics (TSS) and had the lowest Akaike Information Criteria (AIC). Rhinoceros occurrence was best explained by (a) Distance from grassland, (b) Distance from forest, (c) Maximum temperature of warmest month (d) Annual Precipitation and, (e) Distance from water (Table 1). As expected, species response curve showed a decline in habitat suitability with increasing distance from grasslands (Fig. 1c). Rhinoceros were unlikely to occur at distances > 2 km from grasslands. Distance from grassland habitat explained the maximum variation (69%) in the rhinoceros distribution model. Rhinoceros occurrence declines rapidly from forest edge (Fig. 1d) which contributed 16% to the model. A temperature range between 32 to 40 °C during the warmest months of the year governed occurrence of rhinoceros (Fig. 1e). This covariate (Maximum temperature of the warmest month) explained 8.2% of the variation in the data in the best model. Rhinoceros occurred where annual rainfall exceeded 1700 mm and the probability of occurrence increased with increase in rainfall up to 4000 mm (Fig. 1f). Rhinoceros occurrence probability declined sharply with increasing distance to water up to 5 km, after this distance there was high variability in the response curve, This covariate contributed the least to the model at 1.9% (Fig. 1g).

**Table 1:** Covariates, their contribution to explaining species occurrence, and their sources.

Variables	Rhino					Buffalo					Source
	Training AUC	Test AUC	TSS	Kappa	AIC	Training AUC	Test AUC	TSS	Kappa	AIC	
**Climate**											
Maximum temperature of warmest month (MXT)	0.92	0.87	0.54	0.07	10,734.8	0.88	0.84	0.41	0.00	1762.0	Worldclim website
Annual precipitation (AP)	0.89	0.85	0.69	0.05	11,501.6	0.85	0.82	0.35	0.00	1792.4	Worldclim website
Minimum temperature of coldest month (MIT)	0.84	0.84	0.50	0.02	11,723.6	0.88	0.83	0.54	0.00	1749.4	Worldclim website
Precipitation of driest quarter (PQ)	0.88	0.85	0.53	0.03	11,356.6	0.82	0.75	0.19	0.00	1801.5	Worldclim website
Precipitation of wettest month (PW)	0.78	0.71	0.70	0.00	12,135.7	0.87	0.80	0.36	0.00	1780.5	Worldclim website
**Abiotic habitat**											
Elevation (E)	0.87	0.82	0.40	0.01	11,654.3	0.87	0.82	0.42	0.00	1763.3	SRTM Data
Distance from water (DW)	0.70	0.70	0.34	0.01	12,391.2	0.77	0.72	0.56	0.01	1844.9	NDWI & MNDWI from LandSat 8
**Biotic habitat**											
Distance from grassland (DGL) and ground validation	0.92	0.89	0.20	0.00	10,810.3	0.92	0.91	0.68	0.02	1648.9	Digitized using LandSat 8, Google Earth Pro
Distance from forest (DF)	0.78	0.79	0.40	0.01	11,954.1	0.73	0.72	0.29	0.00	1837.3	GlobCover (2009)
Pre-monsoon NDVI (PRNDVI)	0.71	0.73	0.13	0.00	12,368.2	0.74	0.73	0.44	0.00	1897.3	MODIS Vegetation Index Products
Post-monsoon NDVI (PONDVI)	0.79	0.76	0.37	0.01	12,056.4	0.73	0.65	0.12	0.00	1900.6	MODIS Vegetation Index Products
Difference in pre-monsoon and post-monsoon NDVI (DNDVI)	0.70	0.63	0.15	0.00	12,397.4	0.66	0.62	0.06	0.00	1873.3	MODIS Vegetation Index Products
**Anthropogenic disturbance**											
Human footprint Index (HF)	0.74	0.76	0.27	0.03	12,159.0	0.72	0.79	0.00	0.00	1844.6	Last of the Wild Project, Version2, 2005 (LWP-2)
Distance from PA (DPA)	0.93	0.93	0.00	0.00	10,590.2	0.91	0.91	0.00	0.00	1636.7	Protected Planet website
**Combined climate, biotic and abiotic habitat parameter, and anthropogenic disturbance**											
PW + DGL + DF + DW + PONDVI	0.95	0.95	0.77	0.09	10,376.9	0.97	0.98	0.82	0.02	1594.2	
PW + DGL + DF + DW	0.95	0.94	0.79	0.10	10,581.7	0.96	0.99	0.850	0.016	1641.6	
PQ + DGL + DF + DW	0.97	0.96	0.79	0.12	9620.7	0.97	0.94	0.66	0.03	1567.2	
MXT + AP + DGL + DF + PONDVI + DW	0.96	0.95	0.82	0.15	10,003.3	0.968	0.958	0.75	0.045	1578.26	
**MXT + AP + DGL + DF + DW**	**0.97**	**0.96**	**0.84**	**0.11**	**9491.1**	0.96	0.96	0.68	0.04	1502.0	
**AP + DGL + DF + DW**	0.97	0.95	0.80	0.12	9757.3	**0.96**	**0.94**	**0.854**	**0.03**	**1550.4**	

Here AUC- Area Under Curve, TSS- True Skill Statistics and AIC- Akaike Information Criteria.

The best models (bold and underlined) were most parsimonious and ecologically meaningful models with high AUC, best TSS and small AIC values.

#### Swamp buffalo

**Figure 2:**
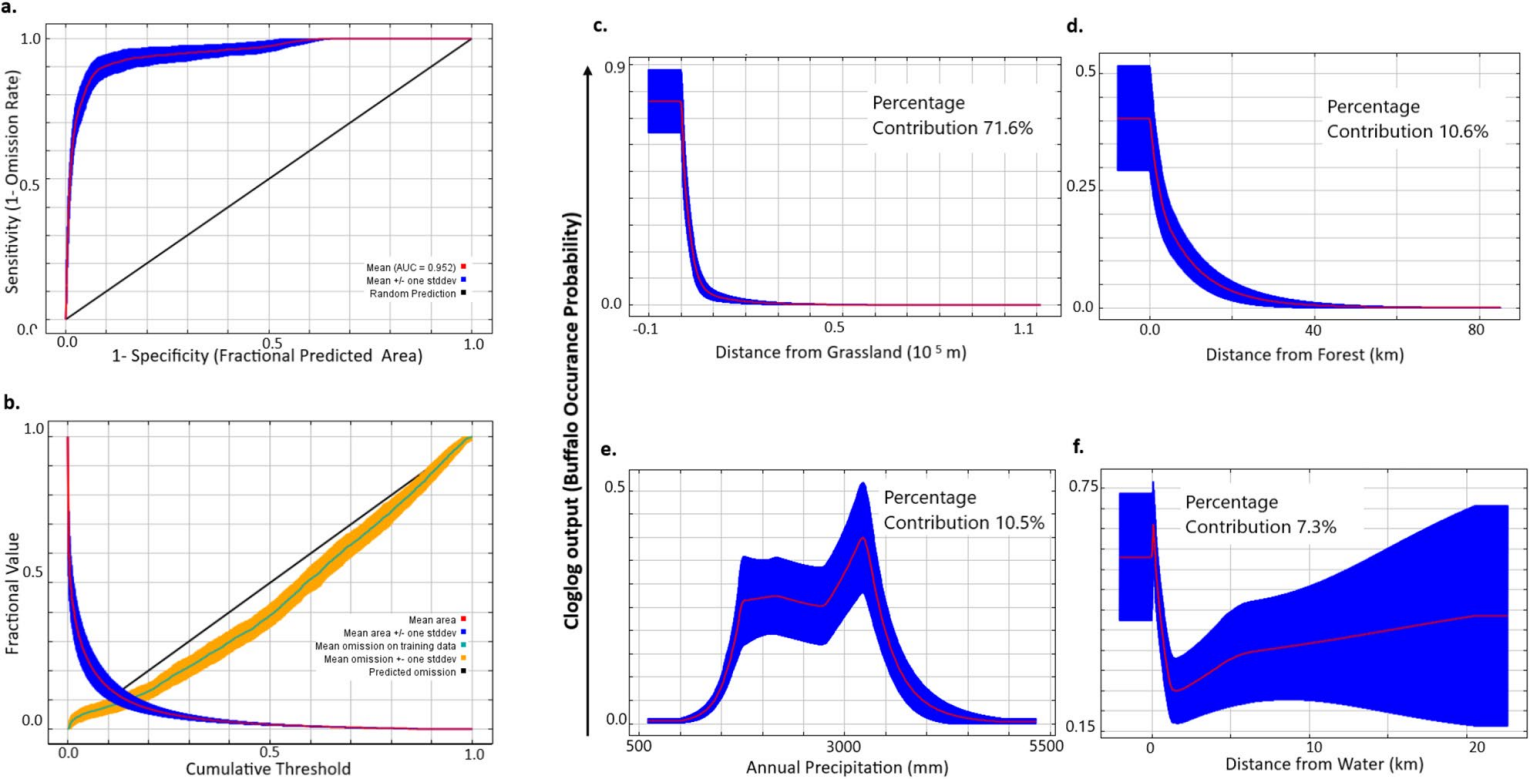
(**a**) Receiver Operator Curve for assessing model fit, (**b**) Omission/Commission analysis for model accuracy of classifying test data. Response curves obtained from 100 bootstrap runs of modeling distribution of wild swamp buffalo in Maxent to (**c**) distance to grassland, (**d**) distance to forest, (**e**) Annual precipitation and (**f**) distance to water for the Terai-Brahmaputra floodplains landscape.

The Buffalo model had a good fit with an AUC of 0.95 (SE 0.0027) but the predictive ability based on 100 bootstrap runs of omission/commission analysis with 20% test data (Fig. 2a,b) was not as good as that for rhinoceros. The TSS was the best for this model while the AIC was second best (Table 1). However, since this model had a good fit and made ecological sense supported by TSS we use it as the best model. The covariates of the best buffalo model were (a) Distance from grassland, (b) Distance from forest, (c) Annual Precipitation and, d) Distance from water (Table 1). Distance to grassland had the highest contribution to the model. Buffalo occurred in the proximity of grasslands and were not likely to be found beyond 1 km distance from grasslands (Fig. 2c). The model showed high buffalo habitat suitability at forest edges with low probability with increasing distances from forests (Fig. 2d). Areas having annual rainfall above 1500 mm were preferred (Fig. 2e)*.* Habitat suitability for buffalo declined rapidly with increasing distance from water for up to 2 km after which habitat was unsuitable with high variability in model predictions (Fig. 2f).

### Population habitat viability analysis (PHVA)

#### Greater one-horned rhinoceros

PHVA for rhinoceros revealed small populations (K ≤ 10) could not persist (Table 2, Scenarios 1–4 and Table S1). Medium sized populations (K = 20–30) are shown to be viable when initial reintroductions are undertaken with > 8–10 rhinoceros and sites are occasionally supplemented, but these populations would not withstand poaching (Table 2, Scenarios 5–11 and Table S1,). Populations with K ≥ 50 have better chances of survival which increases with supplementation. These populations would also withstand low levels of poaching when supplemented at initial stages (Table 2, Scenarios 14 & 15). Populations ~ 100 would survive long-term, even when subjected to a low-level poaching, and are able to retain high levels of heterozygosity even without immigrants (Table 2, Scenario 19–21 and Table S1). Populations with K ≥ 100 are ideal for long-term persistence (Table 2, Scenario 22). A meta-population comprising of Kaziranga, Orang and Laokhowa-Bura Chapori demonstrates a stochastic growth rate of 0.022 (SE 0.019). The extinction probability of that metapopulation is zero and heterozygosity is maintained at ~ 100%.

**Table 2:** Results of 500 simulations of population trajectories over 100 years in VORTEX (9.93) to assess the viability of greater one-horned rhinoceros populations with different scenarios of carrying capacity, poaching, catastrophe, initial population size and supplementation.

Scenario	Carrying capacity	Initial population	Supplementation	Frequency of catastrophes	Frequency of harvest	r (SD)	PE	N	H%
1	10	5(3AF & 2AM)	None	None	None	0.015(0.126)	0.85	5	40
2	10	5(3AF & 2AM)	None	4% flood	None	0.014 (0.127)	0.87	5	44
3	10	5(3AF & 2AM)	2 in 2 years (1AF &1AM) for first 5 years	None	None	0.029 (0.139)	0.84	5	44
4	10	5(3AF & 2AM)	None	4% flood	2 in 5 years (1AF &1AM)	0.021 (0.183)	1.00	0	0
5	20	5(3AF & 2AM)	None	None	None	0.016 (0.096)	0.28	63	12
6	20	5(3AF & 2AM)	None	4% flood	2 in 5 years (1AF & 1AM)	0.023 (0.176)	1.00	0	0
7	20	8(6AF & 2AM)	3 in 2 years (2AF &1AM) for first 6 years	4% flood	2in10 years (1AF& 1AM)	0.020 (0.109)	25	11	67
8	20	5(3AF & 2AM)	2 in 2 years (1AF &1AM) for first 5 years	4% flood	2 in 5 years (1AF &1AM)	0.003 (0.144)	0.96	8	58
9	20	10(7AF & 3AM)	2 in 2 years (1AF &1AM) for first 5 years	4% flood	2 in 5 years (1AF &1AM)	0.011 (0.125)	0.89	8	63
10	30	10(7AF & 3AM)	3 in 2 years (2AF &1AM) for first 6 years	4% flood	None	0.027 (0.076)	0.01	22	77
11	30	8(6AF & 2AM)	3 in 2 years (2AF &1AM) for first 6 years	4% flood	2in10 years (1AF& 1AM)	0.025 (0.087)	0.03	21	78
12	50	10 (7AF &3AM)	None	None	None	0.025 (0.06)	0.00	41	81
13	50	10 (7AF &3AM)	5 (3AF &2AM) every 2 years for first 5 years	None	None	0.035 (0.078)	0.00	42	87
14	50	10 (7AF &3AM)	5 (3AF &2AM) every 2 years for first 5 years	4% flood	2 in 5 years (1AF &1AM)	0.028 (0.84)	0.03	35	85
15	50	10 (7AF &3AM)	5 (3AF &2AM) every 2 years for first 5 years	4% flood	3 in 5 years (1AF &2AM)	0.023 (0.087)	0.03	35	84
16	50	10 (7AF &3AM)	5 (3AF &2AM) every 2 years for first 5 years	4% flood	5 in 5 years (2AF &3AM)	0.008 (0.119)	0.37	26	80
17	75	10 (7AF &3AM)	None	None	2 in 5 years (1AF &1AM)	0.008 (0.116)	0.78	40	75
18	75	10 (7AF &3AM)	5 (3AF &2AM) every 2 years for first 5 years	None	2 in 5 years (1AF &1AM)	0.029 (0.060)	0.00	57	90
19	100	10 (7AF &3AM)	None	None	None	0.027 (0.055)	0.01	86	84
20	100	10 (7AF &3AM)	None	4% flood	2 in 5 years (1AF &1AM)	0.008 (0.091)	0.39	56	78
21	100	10 (7AF &3AM)	5 (3AF &2AM) every 2 years for first 5 years	4% flood	5 in 5 years (2AF &3AM)	0.020 (0.092)	0.12	76	88
22	150	10 (7AF &3AM	5 (3AF &2AM) every 2 years for first 5 years	4% flood	5 in 5 years (2AF &3AM)	0.020 (0.090)	0.16	108	88

Here AF – adult female, AM – adult male, r = growth rate of population, (SD) = standard deviation, N = population size at the end of 100 years, PE = probability of Extinction and H = heterozygosity of the population at the end of 100 years.

#### Swamp buffalo

Small populations (K ≤ 20) exhibit low persistence probability despite supplementation (Table 3, Scenarios 1–4 and TableS2). Medium sized populations (K = 50) could persist with initial reintroduction of > 10 individuals supplemented for a decade, but would not sustain poaching offtake (Table 3, Scenarios 5-9 and Table S2). Large populations (K = 100–200 buffalos) remain viable in settings which experience natural catastrophes, and are also resilient to moderate poaching losses if initial founding population is > 30. Populations are sensitive to the size of founding population and depend upon continued supplementation (Table 3, Scenario 10–16 and Table S2). Areas which could sustain (K) > 250 were ideal for long-term persistence and could tolerate moderate poaching. However, all populations were sensitive to founding population size, and a founding population > 30 is optimal.

**Table 3:** Results of 500 runs of population habitat viability analysis of wild swamp buffalo populations with different scenarios of carrying capacity, poaching, catastrophe, initial population size and supplementation over a period of 100 years.

Scenario	Carrying capacity	Initial population	Supplementation	Frequency of catastrophes	Frequency of harvest	r (SD)	PE	N	H%
1	20	10 (6AF & 4AM)	None	None	None	0.006 (0.156)	0.65	13	34
2	20	10 (6AF & 4AM)	2 (1AF &1AM) every year for first 5 years	None	None	0.013 (0.155)	0.62	14	37
3	20	10 (6AF & 4AM)	2 (1AF &1AM) every year for first 10 years	4% floods + 2% Diseases outbreak	None	0.015 (0.160)	0.67	13	38
4	20	10 (6AF & 4AM)	2 (1AF &1AM) every year for first 10 years	None	2 (1AF & 1AM) every year for 100 years	0.014 (0.196)	1	0	0
5	50	10 (6AF & 4AM)	None	None	None	0.014 (0.129)	0.43	37	54
6	50	10 (6AF & 4AM)	2 (1AF &1AM) every year for first 5 years	None	None	0.023 (0.111)	0.1	39	63
7	50	10(7AF, 3AM)	3 (2AF &1AM) every 2 years for first 10 years	4% floods + 2% Diseases outbreak	2(1AF &1AM) every 15 years for 100 years	0.020 (0.118)	0.11	34	64
8	50	10 (6AF & 4AM)	2 (1AF &1AM) every year for first 10 years	4% floods + 2% Diseases outbreak	None	0.022 (0.112)	0.06	37	66
9	50	10 (6AF & 4AM)	2 (1AF &1AM) every year for first 10 years	None	2 (1AF & 1AM) every year for 100 years	0.007 (0.157)	0.88	29	58
10	100	10 (6AF & 4AM)	None	None	None	0.017 (0.125)	0.43	78	60
11	100	20 (10AF & 10AM)	None	None	None	0.021 (0.091)	0.05	80	72
12	100	20 (10AF & 10AM)	2 (1AF &1AM) every year for first 5 years	None	None	0.024 (0.084)	0.01	82	77
13	100	20 (10AF & 10AM)	2 (1AF &1AM) every year for first 5 years	None	2 (1AF & 1AM) every 2 years for 100 years	0.001	0.58	71	71
14	100	20 (10AF & 10AM)	2 (1AF &1AM) every year for first 5 years	4% floods + 2% Diseases outbreak	2 (1AF & 1AM) every 2 years for 100 years	0.013 (0.135)	0.76	62	71
15	100	30 (20AF 10AM)	2 (1AF &1AM) every year for first 5 years	4% floods + 2% Diseases outbreak	2 (1AF & 1AM) every 2 years for 100 years	0.001 (0.105)	0.36	65	76
16	200	30 (20AF 10AM)	2 (1AF &1AM) every year for first 5 years	4% floods + 2% Diseases outbreak	2 (1AF & 1AM) every 2 years for 100 years	0.008 (0.095)	0.27	134	79
17	250	10 (10AF & 10AM)	None	4% floods + 2% Diseases outbreak	None	0.020 (0.094)	0.08	169	73
18	250	20 (10AF & 10AM)	None	None	None	0.025 (0.084)	0.05	192	75
19	250	20 (10AF & 10AM)	2 (1AF &1AM) every 2 years for first 5 years	4% floods + 2% Diseases outbreak	2 (1AF & 1AM) every 2 years for 100 years	0.018 (0.140)	0.83	139	71
20	250	35 (20AF & 15AM)	2 (1AF &1AM) every 2 years for first 10 years	4% floods + 2% Diseases outbreak	2 (1AF & 1AM) every 2 years for 100 years	0.009 (0.091)	0.25	161	80
21	500	20 (10AF & 10AM)	None	None	None	0.024 (0.089)	0.09	307	74
22	500	35 (20AF & 15AM)	None	4% floods + 2% Diseases outbreak	2 (1AF & 1AM) every 2 years for 100 years	0.001 (0.106)	0.48	212	76
23	500	35 (20AF & 15AM)	5 (3AF &2AM) every 2 years for first 5 years	4% floods + 2% Diseases outbreak	2 (1AF & 1AM) every 2 years for 100 years	0.012 (0.088)	0.22	254	81

Here AF – adult female, AM- adult male, r = growth rate of population, (SD) = standard deviation, N = population size at the end of 100 years, PE = probability of Extinction and H = heterozygosity of the population at the end of 100 years.

### Identifying and prioritizing suitable habitats

**Figure 3:**
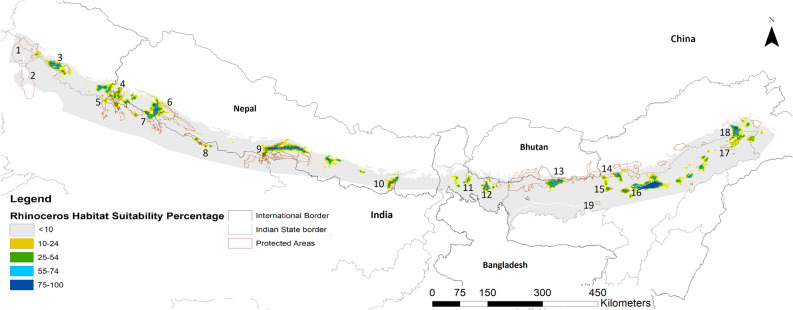
Distribution probability of greater one-horned Rhinoceros across its global historic range in the Terai-Brahmaputra floodplain, where 1-Rajaji NP, 2-Hastinapur WLS 3-Corbett NP, 4- Shukalaphanta NP, 5-Pilibhit TR, 6-Bardia, 7-Keterniyaghat WLS, 8- Sohelwa WLS, 9- Chitwan NP-Valmik TR-Parsa WLS Complex, 10-Koshi Tappu RAMSAR Site, 11- Gorumara WLS, 12- Jaldapara WLS, 13- Manas-Royal NP-Manas NP Complex, 14-Sonai Rupai WLS, 15- Orang TR, 16- Kaziranga NP, 17-Dibru Saikhowa WLS, 18- D’Ering Memorial WLS, 19- Pobitora WLS. Created in ESRI ArcMap 10.5.1 (https://support.esri.com/en/Products/Desktop/arcgis-desktop/arcmap/10-5-1#downloads).

**Figure 4:**
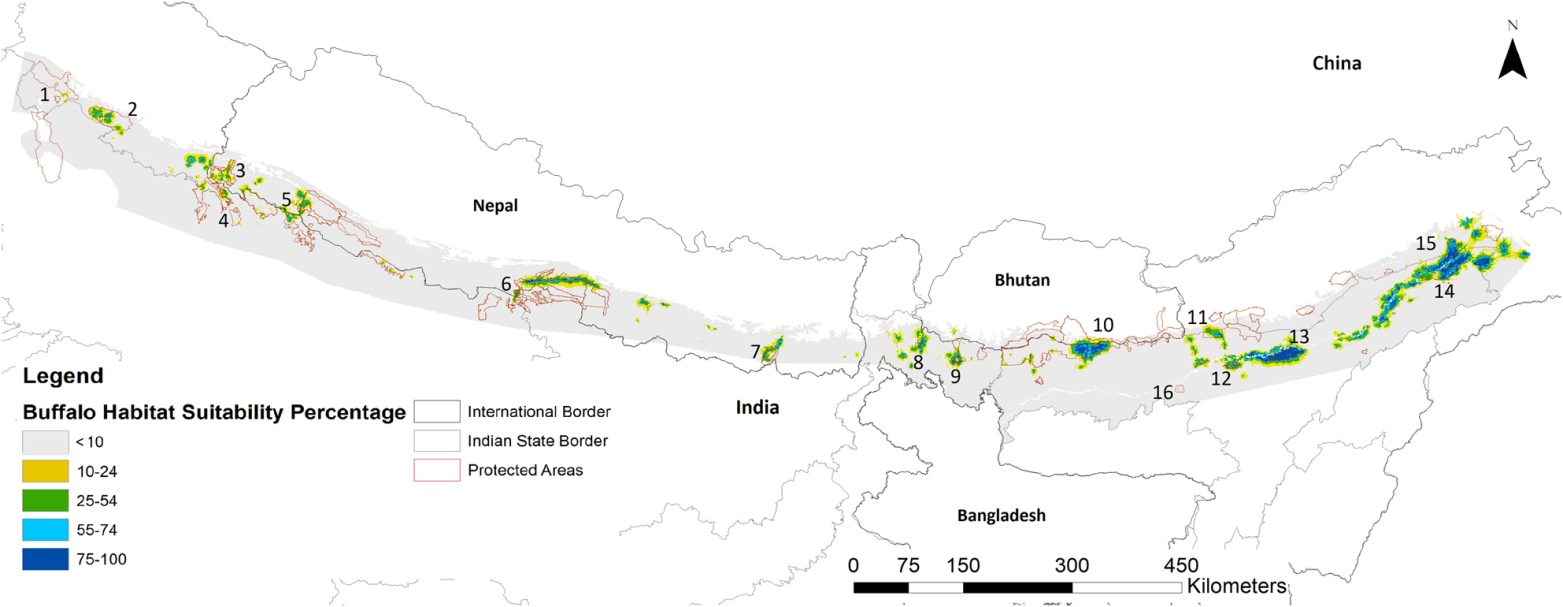
Distribution probability of wild swamp buffalo across the Terai-Brahmaputra floodplain, where 1-Jhilmil Jheel, 2-Corbett NP, 3- Shukalaphanta NP, 4- Kishanpur WLS, 5-Keterniyaghat WLS, 6- Chitwan NP-Valmik TR-Parsa WLS Complex, 7-Koshi Tappu RAMSAR Site, 8- Gorumara WLS, 9- Jaldapara WLS, 10- Manas-Royal NP-Manas NP Complex, 11-Sonai Rupai WLS, 12- Laokhowa-Burachapori WLS, 13- Kaziranga NP, 14-Dibru Saikhowa WLS, 15- D’Ering Memorial WLS, 16- Pobitora WLS Created in ESRI ArcMap 10.5.1 (https://support.esri.com/en/Products/Desktop/arcgis-desktop/arcmap/10-5-1#downloads).

The species distribution probability asc. layer obtained as the median from 100 Maxent bootstrap runs were exported to Arc GIS 10.5 to produce probability maps for rhinoceros (Fig. 3) and buffalo (Fig. 4), showed reasonable extents of suitable habitat outside of these species’ current range. Maps from conservative estimates of 95% lower limits also identified substantial patches of suitable habitat for reintroductions (Fig S1). Maximum training sensitivity plus specificity cumulative threshold values for 100 bootstrap runs for the rhinoceros model was less variable (13.95 ± SE 0.196; range 9.6–18.5) when compared to that of the buffalo model (19.77 ± SE 0.88; range 1.7–54.7). Maxent identified 11 and 7 habitat patches for rhinoceros and buffalo respectively that could sustain > 50 and > 100 individuals of each species outside of the current range (Table 4). After prioritizing these sites based on legal status, protection, management efficacies and minimal potential for human conflict we identified Corbett NP and Valmiki Tiger Reserve (TR) as top priority sites for rhinoceros reintroduction. For buffalo reintroductions the PA complex of Chitwan NP-Valmiki TR-Parsa Wildlife Sanctuary (WLS) and Bardia NP-Shuklaphanta NP-Dudhwa NP-Katerniagath WLS-Pilibhit TR were top priority (Table S3). While, Bardia NP and Shuklaphanta NP in Nepal and Dudhwa NP, Manas NP in India would benefit from Rhinoceros supplementation (Table 4 & Table S3).

**Table 4:** Suitable habitats for rhinoceros and buffalo, their legal status, current/potential population that each site can sustain and possibility of metapopulation structure.

Protected area name	State, country	Protected status	Rhinoceros						Buffalo						
			Habitat size (95% upper CI) Km^2^	Habitat size (median) km^2^	Habitat size (95% lower CI) km^2^	Density (per km^2^)	Current population	Potential rhino population (minimum)	Habitat size (95% upper CI) Km^2^	Habitat size (median) km^2^	Habitat size (l95% lower CI) km^2^	Density (per km^2^)	Current population	Potential population (minimum)	
Jim (Corbett)	Uttarakhand, India	National Park, Tiger Reserve	375	254	177	1	0	254(177)	243	134	27	1	0	134(27)	Isolated population
Katerniagath	Uttar Pradesh, India	Wildlife Sanctuary	143	136	116	1	0	136 (116)	184	167	145	1	0	167 (145)	Meta population
Pilibhit	Uttar Pradesh, India	Tiger Reserve	222	124	31	1	0	124(31)	156	63	3	1	0	63(3)	
Dudhwa	Uttar Pradesh, India	Tiger Reserve	172	72	42	1	~ 60	72 (42)	171	35	6	1	0	35 (6)	
Kishanpur	Uttar Pradesh, India	Wildlife Sanctuary	7	1	0	1	0	1	25	2	0	1	0	2	
Shuklaphanta	Nepal	National Park	324	279	145	1	~ 30	279 (145)	279	173	32	1	0	173 (32)	
Banke	Nepal	National Park	33	1	0	1	0	1	0	0	0	1	0	0	
Bardia	Nepal	National Park	387	304	207	1	~ 15	304 (207)	106	67	54	1	0	67 (54)	
Valmiki	Bihar, India	Tiger Reserve	52	44	33	1	Occasional immigrants from Chitwan	44 (33)	35	27	10	1	0	27 (10)	Meta population
Chitwan	Nepal	National Park	731	619	502	1.4^α^	~ 600	867 (703)	689	461	248	1	Recently introduced population (< 20)	461 (248)	
Parsa	Nepal	Wildlife Reserve	43	38	34	1	0	38 (34)	49	29	17	1	0	29 (17)	
Laukhowa-Bura Chapori Complex	Assam, India	Wildlife Sanctuary complex	95	95	75	1	1	95 (75)	97	95	58	1	0	95 (58)	Meta population
Orang	Assam, India	Tiger Reserve	63	51	28	2	~ 30	102 (56)	65	61	27	1	0	61 (27)	
Kaziranga	Assam, India	National Park	368	368	368	6^α^	~ 2400	2208	368	368	367	4.2 ^α^	~ 1600	1546	
Gorumara	West Bengal, India	National Park	43	33	25	0.6 ^α^(2)	~ 50	66 (50)	70	51	28	1	0	51 (28)	Isolated population
Jaldapara	West Bengal, India	Wildlife Sanctuary	155	134	122	1.7 α	~ 230	227 (207)	145	103	76	1	0	103 (76)	Isolated population
Koshi Tappu	Nepal	Wildlife Reserve, RAMSAR Site	86	76	50	1	0	76 (50)	124	85	34	1.4 ^α^	~ 250	250	Isolated population
Royal Manas	Bhutan	National Park	26	17	8	1	0	17 (8)	216	138	56	1		138 (56)	Meta population
Manas	Assam, India	National Park	604	532	448	1	~ 30	532 (448)	901	670	537	1	> 500	670 (537)	
D'Ering Memorial (Lali)	Arunachal Pradesh, India	Wildlife Sanctuary	196	194	192	1	0	192	196	195	186	1	Unknown < 100	195 (186)	Meta population
Dibru Saikhowa	Assam, India	National Park	221	169	84	1	0	169 (84)	361	328	270	1	> 100	328 (270)	
Sonai Rupai	Assam, India	Wildlife Sanctuary	154	139	127	1	0	139 (127)	168	143	97	1	0	143 (97)	Isolated population
Buxa	West Bengal, India	Tiger Reserve	21	10	4	1	0	10 (4)	8	0	0	1	< 20		Isolated population
Rajaji	Uttrakhand, India	National Park	171	76	34	1	0	876 (34)					0		Isolated population
Sohagibarwa	Uttar Pradesh, India	Wildlife Sanctuary	12	6	0		0	0	19	5	0	1	0	5	Isolated population
Sohelwa	Uttar Pradesh, India	Wildlife Sanctuary	165	109	74	1		109 (74)	87	26	3	1	0	26 (3)	Isolated population
Nameri	Assam, India	Tiger Reserve	72	12	0	1		12	6	0	0		0	0	Isolated population
Gibbon	Assam, India	Wildlife Sanctuary	2					0					0	0	Isolated population
Sonanadi	Uttarakhand, India	Wildlife Sanctuary	254	233	207	1		233 (208)	246	170	67	1	0	170 (67)	Isolated population
Jhilmil Jheel	Uttarakhand, India	Conservation Reserve				1		0	3	0	0		0	0	Isolated population
Pobitora*	Assam, India	Wildlife Sanctuary	7	4	3	2.6 ^α^	~ 100	*102	6	4	2	2.6 ^α^	~ 100	*100	Isolated population

*Pobitora has extant population of ~ 100 Greater *one-horned* Rhinoceros and ~ 100 Buffalos.

α Actual know crude density at extant sites.

## Discussion

Reintroductions offer great potential for species recovery when following scientifically-informed strategies and relevant risk assessment^22^. Our evaluation of potential reintroduction sites and extant populations addresses these aspects, although any subsequent site-specific plans must also consider IUCN guidelines. Several identified patches sit inside Protected Areas (PAs) with some ready to receive animals, since threats have been addressed and park management capacity exists. We highlight the most feasible actions needed at potential sites. Reintroduction would create safety-net populations for megaherbivores and restore their ecosystem-engineering role, benefitting endangered grassland specialists including pygmy hog *(Porcula salvania)*, hispid hare *(Caprolagus hispidus)*, swamp deer *(Rucervus duvaucelii*), hog deer (*Axis porcinus*), and Bengal florican *(Houbaropsis bengalensis*).

We used a well-established modeling approach of Maxent models^23^ with presence locations obtained from all extant populations to identify potential habitats. Since the modelling space defined in Maxent was clipped within limits of current species’ distribution (elevation, temperature, precipitation), the response curves of these variables should be viewed accordingly; we did not model suitable range in Maxent, but rather the high preference habitats within this suitable range. Additionally, covariates used in the final model were uncorrelated, so the response curves of each covariate are similar when compared to individual and cumulative contribution with all other covariates (Fig. S2). By capturing variability in model-based inference on 100 bootstrap runs for 95% lower limits, inferences remain conservative.

All our presence locations were within PAs (since surviving populations exist only in PAs), so the metric ‘distance to PA’ would overpredict potential for occurrence in most PAs, we therefore refrained from using this covariate in our final models. Instead we accounted for legal status of habitats after the SDM modelling process during decision-making. Human Footprint Index did not conform to a priori hypothesis and was not used after exploratory analysis, since most of the fertile floodplain habitats outside the realm of legal protection were already occupied for human land use. Species presence locations were often juxtaposed with pixels having high Human Footprint values due to hard PA boundaries, making it ecologically uninformative. Variables such as ‘distance from natural grasslands’ and ‘distance from forest’ better captured negative influences of human impact compared to Human Footprint Index and were aligned with a priori expectations.

Often the test data of species occurrence used to validate models are in close proximity to species presence points used for model building, causing spatial autocorrelation^24^ that inflates the accuracy (AUC) of the model^25^. In both megaherbivore models geographic clustering of training and test data may have caused spatial autocorrelation and inflation of AUC values. However, our assertion is that species occurrence data used in our models spans the entire extant geographic range of both megaherbivores across the entire spectrum of covariate space occupied by each species. We use only one presence location per one km^2^ pixel (as explained by Philips et al. 2017^85^), and use the bias correction file option in Maxent to address this limitation of training data^26^. We also use TSS and AIC to evaluate our models.

Our habitat suitability analysis showed PAs such as Kishanpur WLS, Sohagibarwa WLS and Nameri TR had suitable habitat to support ≤ 10 rhinoceros. Results of PHVA for rhinoceros suggest small populations (≤ 10) had low probability of persistence so these areas are rejected. Suitable habitat identified in Rajaji NP was in the floodplains of the Ganges and its tributaries, an area heavily utilized for religious pilgrimage in proximity to townships of Haridwar and Rishikesh, again unsuitable for reintroduction due to potential human-wildlife conflict.

Although our analysis suggests that Valmiki itself could support only a small population (35–40 individuals), we recommend reintroduction, since Valmiki receives natural immigrants from Chitwan (Nepal), so offers probability of long-term metapopulation persistence. That said, investment to re-route the railway line passing through prime rhinoceros habitat is necessary, and is being considered by the Bihar Government (Valmiki Park Director 2018, Personal Communication). An increase in law enforcement and reduction of human activities are also required, although Valmiki has clearly improved its protection regime in recent years as evidenced by an increasing tiger population^27^, a species vulnerable to poaching^28^.

Encouragingly, existing rhino ranges in Shuklaphanta, Bardia, and Orang have potential to sustain higher numbers. Supplementation would be possible if there is investment in protection, habitat management, and reduction of anthropogenic pressures and human-rhinoceros conflict. The same is needed for existing reintroduced populations in Dudhwa and Manas which would also benefit from supplementation to mitigate inbreeding^29,30^. Manas NP could support around 500 rhinoceros, but the current population is restricted to low densities in the eastern part of the PA. Manas park managers were reluctant to increase rhinoceros numbers due to a lack of infrastructure to ensure protection (Manas Park Director 2018, Personal Communication). Concerns are well-founded; the region suffers socio-political unrest and Manas previously lost rhinoceros to poaching during civil unrest in the 1990′s^18^. Significant investment in training, vehicles, weapons, and M-STrIPES patrolling tool is required for recovery of rhinoceros (and tiger) numbers in Manas NP. Royal Manas NP (Bhutan) is contiguous with Indian Manas NP and could independently support 20 rhinoceros, but managers have similar concerns about resources to protect reintroduced animals (Royal Manas Park Director 2018, Personal Communication). Social instability makes translocation to Manas or Royal Manas difficult.

Our analysis suggests that Corbett TR can sustain a population of > 150 rhinoceros (Table 4). Site evaluation and habitat studies (including grassland food-source species like *Saccrum, Imparata, Arundo* and *Vetivaria)*^31^ suggest that Corbett is suitable, with ample surface water (rivers, reservoirs, pools)^32^ plus sufficient grassland patches interspersed with forest cover, to support mixed foraging and cover during calving and in winters^33^. Habitat management such as artificial wallows may be required, since the largely *Bhabhar* landscape lacks muddy swampland and oxbow lakes of the *Terai.* The level of protection in the park is high with managers capable of handling reintroduction^34^. A key benefit of Corbett over other PAs is political stability in Uttarakhand, including in and around the PA (in contrast to Assam and Nepal^18^). The large valleys of Corbett TR offer ideal habitats for rhinoceros, bounded by the lower Himalayas (north), Shivalik Hills (south) and the Ramganga Reservoir, retaining rhinoceros within the PA, so limiting conflict.

Laukhowa-Bura-Chapori complex, proposed for reintroductions by Rhino Vision 2020^36^, was also identified by our study, however considerable conservation investments are needed before reintroduction (Fig. S3). Major interventions needed include (a) reduction of cattle grazing, (b) grassland management and weed control, (c) protection (guards, weapons, law enforcement training, vehicles, and infrastructure). The site is of particularly significance^27^, as it connects habitat along the Brahmaputra river for rhinoceros, elephants, buffalo and tigers dispersing from Kaziranga. Our PHVA analysis shows that if rhinoceros in Orang TR, Kaziranga NP and Laokhowa-Bura Chapori WLS complex are managed as a metapopulation they will persist even in the small reserves of Orang and Laokhowa-Bura Chapori for the long-term. Although Dibru Saikhowa WLS (Rhino Vision 2020 site)^36^ and D’Ering offer extensive habitat, both are affected by seasonal agriculture encroachment and high livestock density. Effort to remove or reduce those threats would enable these island sanctuaries to hold an estimated > 100 rhinoceros each.

One potential metapopulation includes the complex of Bardia and Shuklaphanta of Nepal across the international border to the Indian complex of Dudhwa, Katerniagath, and Pilibhit along the Khata Corridor, Lagga-Bagga WLS and flood-plains of Sharda river^37^. Rhinoceros sometimes transit these PAs, however new proposed border roads will create further obstacles to transboundary wildlife movements. Metapopulation connectivity would benefit other species like (tigers, elephants, buffalos), reducing the need for supplementation.

Corbett is isolated with no adjacent rhinoceros or buffalo populations, however its large carrying capacity would make a population viable if genetically diverse founders are selected, supported by supplementation. Hastinapur WLS in Uttar Pradesh^38^ and Surai Range in Uttarakhand were previously considered for rhinoceros, but our analysis rejects both. Hastinapur WLS lacks forest cover (essential for thermoregulation and calving) and is bordered by agriculture and high human density. The Surai range, although *Terai* habitat, is small and suffers human encroachment.

We have modelled supplementation for a period of 5–10 years, a time period coinciding with most government funded projects. If however, supplementation continues intermittently over the long-term it would dramatically improve persistence and genetic variability of populations. Our population estimates from model-based computations for Chitwan and Kaziranga match the current estimates of rhinoceros at these sites^15^ which suggests that extant populations are nearing carrying capacity and can serve as source populations^39^. Pobitora WLS, which has close to 100 rhinoceros and a sizable buffalo population, was not identified as good habitat in our results. This shows the limitations of model-driven inferences, which may fail to account for outliers resulting from species’ behavioral plasticity or exceptional tolerance by local people. Pobitora is unique, in being imbedded in a human dominated landscape with minimal availability of optimal conditions, yet rhinoceros and buffalo persist in this remnant (39 km^2^) due to community tolerance (Fig. S5).

Our results show (somewhat unexpectedly considering illegal demand for rhinoceros horn) that buffalo are more prone to extinction than rhinoceros, which is actually congruent with the IUCN Red List^40^ which categorises the rhinoceros as vulnerable and buffalo as endangered. Buffalo are rarely poached commercially, but our results suggest that even small off-take due to bush-meat demands would be a major cause of concern. Our PHVA results for buffalo are likely conservative since studies on demographic parameters of wild buffalo are sparse and potentially biased towards higher mortality estimates.

Many buffalo populations of Northeast India are either too small or possibly hybridized with domestic buffalo (derived from swamp buffalo)^16^. In western regions buffalo are largely locally extinct including in locations which could potentially sustain populations. It would be prudent to reintroduce *Bubalus arnee* into suitable western sites, since domestic buffalo in those localities are derived from river buffalos (*Bubalus bubalus*)^41,42^, presenting less risk of hybridization.

Priority sites for buffalo reintroduction in the subcontinent are Chitwan NP-Valmiki TR complex and Shuklaphanta-Bardia-Dudhwa-Katerniagath-Pilibhit complex since both could sustain > 400 individuals. PAs like Sonai Rupai, D’Ering, Dibru Saikhowa, Jaldapara, and Royal Manas could each hold more than 100 individuals, with the potential to be managed as metapopulations. However, bushmeat hunting persists in northeastern India^43^, which poses a major threat to wild buffalo. Higher densities of buffalo may not be possible until human pressures are reduced and management of invasive plants is undertaken. The model-inferred median population of buffalo sustainable in Corbett NP is about 130 individuals. However, there was high level of uncertainty in our model-based estimates for Corbett (Table 4), which we believe was due to the sampling of presence locations being limited to extant populations in the far east region, which experiences different bioclimatic conditions to Corbett in the western *Terai*.

The reintroduction of buffalo could act as a test-bed for rhinoceros reintroductions, enabling establishment of management protocols, enhancing staff experience and ensuring effective future operations for rhinoceros. Reintroduction of buffalo to Dudhwa should be feasible since park managers already have protection and monitoring systems in place following experience of managing reintroduced rhinoceros. Chitwan NP in Chitwan-Parsa Complex of Nepal (a high potential site for buffalo reintroduction in this study) has seen recent buffalo reintroduction, which has a good chance of establishment due to habitat quality, local people’s awareness of ecosystem services, experience of megaherbivores and economic benefits from wildlife tourism^44^.

Knowledge of population genetics must inform any reintroduction. For buffalos, hybridization with domestic buffalo is a key factor, and animals of pure wild lineage are needed as founders for reintroduced populations. Possibly the last pure representatives of *Bubalus arnee* are around 50 buffalo surviving in central India (Udanti-Sitanadi TR, Indravati, in Chhattisgarh and Sironcha in Maharashtra)^45,46^. According to our PHVA models, this Central Indian population suffers a high probability of extinction. The animals reside in a zone of high political unrest, making local conservation difficult^47^. Efforts to rescue these buffalo is vital, including taking animals into captivity for conservation breeding, or translocation.

An important aspect highlighted by our SDM was that both the greater *one-horned* rhinoceros and the wild swamp buffalo have very narrow tolerance of temperature and precipitation (Fig. 1e,f; S5). This has serious implications for persistence of populations under climate change. Current populations of both species are limited to PAs largely surrounded by human land-use, making natural range-shifts unlikely^48^, so conservation policy and strategy must address this limitation by managing metapopulations. Western (Corbett NP) and central terai (Dudhwa-Katerniagath-Bardia) are at higher elevations compared to the Brahmaputra plains and could provide refuges in the advent of climate change.

Several factors limit the potential for reintroducing species in their historic range, including loss and degradation of habitat and competition with human interests. For rhinoceros, even in areas with good habitat and least potential for conflict with humans, managers were reluctant to reintroduce the species, due to resource demands and likely media and political pressure in the face of potential losses of animals. Suitable habitats and management are insufficient for protecting species with high value in illegal wildlife markets; only changes in human perceptions, values, and strict law enforcement will address illegal demand^49^.

We advocate the establishment of safety-net populations across a varied geographical range, and our recommendations address the uncertainties associated with climate change. Continued anthropogenic pressures mean that maintaining effective ecosystems will require balanced species assemblages. Reintroduction and supplementation of viable populations of keystone species are important tools for sustaining and enhancing vital functions in critical natural systems.

## Materials and method

### Field sampling

Field sampling was undertaken to obtain extant rhinoceros and buffalo locations (from India, Nepal and Bhutan) for modelling habitat suitability and assess the reintroduction potential of identified sites. The current extant and potential reintroduction sites were assessed for level of protection, anthropogenic pressures, size, and quality of available habitat for the target species through site visits and interviews with wildlife managers. The interviews addressed managers’ perception of management strengths and weaknesses, and attitude towards reintroduction/supplementation of both megaherbivores (Table S3). All interviews were conducted after obtaining informed consent of the respondents. Prior ethical approval for the study was granted by the School of Anthropology and Conservation, University of Kent, UK, (Reference ID-48-PGT-17/18) following the standards of the American Anthropological Association. All research was conducted under the laws, rules, and regulations of each country. Interviews were conducted in English since all wildlife managers interviewed were conversant with English. Information from site visits and interviews were used to evaluate sites that were selected by SDMs for practicality of reintroductions and potential establishment of populations.

### Species distribution modelling

Several approaches to modelling SDM are available for data typ used in this study i.e. Presence vs. Background points. These include like (1) Ecological niche factor analysis (ENFA)^50^, (2) Genetic algorithm for Rule-set prediction (GARP)^51^ (3) Maximum Entropy models (Maxent)^53^. Earlier researchers have compared the performance of these approaches and found that Maxent models outperform the other approaches^52^. We used Maximum Entropy Species Distribution Modelling in Maxent (Version 3.4.1)^53,54^ for modelling potential habitat of rhinoceros and buffalo . Maxent uses machine learning to develop relationships from known species occurrence and background data with ecologically meaningful spatial environmental covariates^54^. The program subsequently predicts potential distribution of species from covariate relationships across modelled space^53^.

#### Species occurrence data

Species occurrence information from extant populations of both species was obtained by direct sightings with coordinates recorded by hand-held GPS, camera trap photo-capture locations across the Terai-Brahmaputra floodplains in India^27^, and from locations of satellite-GPS-radio-collared rhinoceros in Chitwan NP^14^. To avoid spatial autocorrelation and oversampling, we picked only one location from an area of 1 km^2^^55^. A total of 358 rhinoceros locations and 78 buffalo locations were used for Maxent modelling. Historical records of occurrence were not used since many covariates used in our models have changed since historical times. Furthermore, our modelling objective was to identify extant available habitats and not historical species range.

#### Eco-geographical variables

The following covariates were used as predictive variables for SDM:

*Distance from grassland*

Grasslands of the study area were digitized using unsupervised and supervised classification^56^ in ESRI ArcMap 10.5.1 (Environmental System Research Institute 2016) using LandSat 8 imagery^57^and corrected using Google Earth. We ground-validated grassland locations by obtaining coordinates from a hand-held GPS device (Fig S6). Of the 141 ground validation points 125 were correctly classified resulting in an accuracy of 88.6%. Euclidean Distance tool in ArcMap 10.5.1 was used to calculate the distance of each pixel from grassland polygons at a resolution of 30 m. Both rhinoceros and buffalo can be considered as grassland dependent species^46,58^. A priori we expected both rhinoceros and buffalo occurrence probability to be high within, and on the edge of, grassland habitats and to decline rapidly with increasing distance to grasslands.

*Distance from forest*

Data on forest cover was obtained from GlobCover (2009) at a spatial resolution of 300m^59^. Euclidean Distance of each pixel to a forest patch was computed using ESRI ArcMap (10.5.1). Both study species inhabit grassland-forest mosaic habitats^46,58^ . We expected both species to have high occurrence in forest-edge habitat with a declining response for increased distance to forests.

*Distance from water*

Spectral indicators such as Normalized Difference Water Index and Modified Normalized Difference Water index were used to classify waterbodies (> 30m^2^) from LandSat 8 imagery^60^. However, smaller water bodies which are also used by these species could not be detected by satellite imagery and therefore not accounted for in our models. Euclidean Distance to water for each pixel was calculated using ArcMap 10.5.1. Both rhinoceros and buffalo need ample access to surface water and spend a lot of their time wallowing for thermoregulation^61^. In Subedi’s^33^ study all radio-collared rhinoceros’ locations in Chitwan NP were within 1.8 km distance from water bodies. An essential element of rhinoceros and buffalo habitats are flowing rivers, pools and oxbow lakes. Studies on behaviour of both species show the use of pools and oxbow lakes for wallowing especially in the dry season^45,62^. Our expectation was high occurrence in proximity to water with a rapid decline in species occurrence with increasing distance from surface water.

*Normalised Difference Vegetation Index (NDVI)*

NDVI composites were derived from Moderate Resolution Imaging Spectroradiometer (MODIS) data, which was obtained from online data pool, developed by NASA Land Processes Distributed Active Archive Centres. MODIS products are available at spatial resolution of 250 m with 16-day interval cycle. Use of NDVI for species modelling enable better predictions when used alongside other ecological variables^63^. In this study, three values of NDVI were used (1) Pre-Monsoon NDVI (March–April), which indexes canopy cover of the driest months, (2) Post-Monsoon NDVI (October–November) which characterise maximum cover, and (3) Difference in NDVI of Post-Monsoon and Pre-Monsoon which indicates monsoonal flush of annuals compared to more perennial canopy cover. We expected rhinoceros and buffalo to have a positive response in occurrence probability to higher pre-monsoon NDVI as these pixels would be correlated with forage availability in resource lean summers. Species occurrence response to NDVI difference was expected to increase with increasing difference, as higher difference to reflects fresh flush of nutritive forage^64,65^.

*Geo-climatic data*

Climatic data are commonly used for modelling species distribution^60,61,66^. Both species prefer wetter habitats and moderate climate^33,46^. The modelling extent was clipped by the limits of species occurrence recorded for elevation, temperature and rainfall^67^. Data on (i) annual rainfall (b15, layer clipped for annual rainfall above 1000 mm), (ii) precipitation of the driest quarter (b17), (iii) maximum temperature of the hottest month (b4, layer clipped below 45 °C temperature), (iv) minimum temperature of the coldest month (b6, layer clipped above 0 °C temperatures) and (v) precipitation of the wettest month (b13) were obtained from Worldclim website^68^ at a resolution of 0.5 km^2^. We hypothesized that both species would show a parabolic or peaked response to rainfall and temperature, with a skew toward higher rainfall and a peak at moderate temperatures.

Elevation plays a major role in the distribution of both the megaherbivores as they prefer flatter and less rugged terrain^9^. Ecological studies on rhinoceros indicate preferences for low-lying flat habitats^58^. However, for buffalo, studies have recorded presence up to 1000 m elevation, so we clipped our modelling extent to elevation below 1000 m. We expected a sharp decline in rhinoceros occurrence with increasing elevation and a similar but shallower decline for buffalo. We used the Digital-Elevation-Model produced by the Shuttle Radar Topography Mission (a joint endeavour by NASA, National Geospatial-intelligence Agency, and German and Italian Agencies)^69^.

*Distance from protected area (PA)*

Both megaherbivores suffer poaching and their current populations are largely restricted to PAs^35,46^. We expected species occurrences to decline as distance from PA increased. PA boundaries were taken from the Protected Planet website (https://www.protectedplanet.net/), the Wildlife database cell of the Wildlife Institute of India and the Project Tiger Directorate. The distance of each pixel from PA was calculated using the Euclidean Distance Tool in ESRI ArcMap (10.5.1).

*Human footprint*

Megaherbivores often conflict with humans due their requirement of vast fertile plains, propensity to crop raiding, damage to human property and lives^9^. Proximity to human settlements also raises the problems of habitat degradation, encroachment, poaching and hunting^70^. We therefore, expected a negative relationship between human footprint index and megaherbivore presence. The Global Human Footprint Index dataset was obtained from Last of the Wild Project^71^, which is the Human Influence Index normalized by biome and realm.

#### Modelling in maxent

The habitat covariates were exported to ArcMap (10.2) to obtain uniform resolution and concordance with grids of 0.83 km^2^ across all layers. A correlation matrix for all environmental layers was computed in Eris ArcMap (10.5.1) (Table S4). The correlation threshold was kept at r >  ± 0.70 to check for redundancy in information. No two correlated variables were used together in a single model.

Maxent operates by developing relationships between known occurrence locations and covariates by comparing them with background locations. Species locations used for training our models were from a restricted part of the modelled space (though all extant species locations were sampled). We therefore used a bias correction file^26^ created using inverse distance weight (IDW) interpolation method in ArcMap (10.5.1) to guide Maxent into picking background locations from space containing occurrence locations^54,72^. This approach would enhance the accuracy of our models by limiting the predictive relationships to be developed from the extent of presence vs background from the same area. We used 80% of presence locations of each species to train Maxent models and the remaining 20% were used to test models. The run type was set at Auto with Linear, Hinge, Quadratic and Product functions were selected in Maxent to model relationships between covariates and species occurrence. The maximum number of background points was set at 10,000. Regularization Multiplier was set at default (1) for both species models. We first explored univariate relationships between covariates and species occurrence. Subsequently, based on the univariate responses, we used the covariate combinations that were ecologically meaningful and represented climate, abiotic and biotic habitat and human disturbance to develop more complex models for our species distribution models.

One hundred bootstrap simulations were run for the best model for both species. The median prediction was used to evaluate sites while 95% upper and lower predictions were used to capture the variability associated with model uncertainty. We report species occurrence probability as spatial maps. To compute patch-areas suitable for reintroduction, we used ‘average of maximum training sensitivity plus specificity cumulative threshold’ to categorise pixels as suitable habitats^23^. We computed 95% confidence intervals on suitable habitats using the threshold value. Covariate selection and model evaluation was based on (a) Receiver Operation Characteristic (ROC) curve^73^, (b) contribution of covariates individually and in ecologically meaningful combinations to explain the variability of the training and test data sets and (c) Omission/Commission analysis of test dataset (d) True Skill Statistics (TSS)^74,75^ and (e) Akaike Information Criteria (AIC)^52,76^ Species response curves to each covariate were examined and ecologically interpreted^77,78^.

Rhinoceros and buffalo were observed to be at high densities in Kaziranga (6 rhinoceros^79^/km^2^; and 3.2 buffalos/km^2^, https://www.kaziranga-national-park.com/wild-buffaloes.shtml) while Chitwan had lower densities of rhinoceros (1.4 Rhinoceros/km^2^)^80^. Gorumara NP in West Bengal includes forest, plantation and patchily distributed grasslands carrying a density of about 0.65 rhinoceros in the PA^81,82^. These differences were likely due to difference in productivity and habitat availability in these PAs; with Kaziranga being more productive and the entire PA being favourable habitat while Chitwan (constituted by *Terai*, *Bhabhar*, and Churia hills) and Gorumara having only parts of their habitat as favourable. We report crude densities for these PA but use a conservative estimate of one individual km^-2^ (and 2 km^-2^ for Orang TR which has habitat similar to Kaziranga) as the potential ecological density of both species for reintroduction sites. Achievable population sizes were computed by multiplying only the suitable habitat area by this density.

### Population habitat viability analysis (PHVA)

We assessed the viability of potential reintroduced and extant populations using PHVA in Vortex 9.93^21^. PHVA allows assessment of persistence of a population over a specified timespan incorporating the stochastic nature of demographic parameters, carrying capacity, environmental fluctuations, genetic variability, and deterministic impacts such as poaching^83^. Information on demographic parameters for rhinoceros was obtained from previous studies in Chitwan^39,58,67^. Demographic parameters for the buffalo were also obtained from literature^16,84^ (Table S5). We modelled populations with carrying capacities (K) ranging between 10–150 for rhinoceros and 20–500 for buffalos to reflect habitat patch sizes estimated through Maxent. Each population was subjected to scenarios with varying sizes of initial population, levels of supplementation, poaching and mortality caused by catastrophe (floods/diseases). Based on population sizes and potential movement between Kaziranga, Orang and Laukhowa-Burachapori, a metapopulation of rhinoceros was modelled. This model provided information on the role of movement across the landscape upon viability of individual populations and the metapopulation. Five hundred simulations for each scenario were run for 100 years. Extinction probability, stochastic growth rate, population size, persistence time and the level of heterozygosity were used as evaluation criteria^39^ for prioritizing potential sites.

### Selection criteria for reintroduction sites and recommendations

We considered habitat clusters of combined minimum size > 50 km^2^ to include only those sites which could hold significant populations by themselves or as a metapopulation. We assessed the identified potential reintroduction sites on: (a) habitat quality and size, (b) potential to sustain reintroduced populations over 100 years, (c) legal status and level of protection available/possible, (d) level of anthropogenic stressors and likely conflict with communities.

## Supplementary Information

Supplementary Information 1.
