# Supplementary material for: Feasibility of reintroducing grassland megaherbivores, the greater one-horned rhinoceros, and swamp buffalo within their historic global range
Source: Sci Rep. 2021 Feb 24;11:4469. doi: 10.1038/s41598-021-83174-4 (PMC7904804; doi:10.1038/s41598-021-83174-4)
Supplement: Supplementary file 1 — Supplementary Information 1. [file 41598_2021_83174_MOESM1_ESM.pdf]

Title: Feasibility of reintroducing grassland megaherbivores, the Greater One-horned Rhinoceros, and Swamp Buffalo within their historic global range.

Authors & Affiliations: Harshini Y Jhala[#1,2], Qamar Qureshi[2], Yadvendradev V Jhala[2] and Simon A Black[1].

[#] Corresponding Author harshiniyj@gmail.com

1-Durrell Institute of Conservation and Ecology,

School of Anthropology and Conservation,

University of Kent,

Canterbury CT2 7NZ,

UK.

2-Wildlife Institute of India,

Chandrabani, Dehradun 248001,

Uttarakhand,

India.

Supplementary Information

Table S1. Results of 500 simulations of population trajectories over 100 years in VORTEX (9.93) to assess the viability of greater one-horned rhinoceros populations with different scenarios of carrying capacity, poaching, catastrophe, initial population size and supplementation.

Scenario	Carrying Capacity	Initial Population	Supplementation	Frequency of Catastrophes	Frequency of Harvest	r (SD)	PE	N	H%
**1**	10	5(3AF & 2AM)	2 in 2 years (1AF &1AM) for first 10 years	None	None	0.36 (0.144)	0.84	5	48
**2**	10	5(3AF & 2AM)	2 in 2 years (1AF &1AM) for first 20 years	None	None	0.055 (0.149)	0.77	5	51
**3**	20	5(3AF & 2AM)	None	4% flood	None	0.012 (0.103)	0.32	11	60
**4**	20	5(3AF & 2AM)	2 in 2 years (1AF &1AM) for first 5 years	None	None	0.026 (0.101)	0.17	13	67
**5**	20	5(3AF & 2AM)	2 in 2 years (1AF &1AM) for first 10 years	4% flood	2 in 5 years (1AF &1AM)	0.007 (0.144)	0.94	9	62
**6**	20	5(3AF & 2AM)	3 in 2 years (2AF&1AM) for first 10 years	4% flood	2 in 5years (1AF &1AM)	0.16 (0.152)	0.93	7	63
**7**	50	10 (7AF &3AM)	None	4% flood	2 in 5years (1AF &1AM)	0.003 (0.096)	0.47	29	76
**8**	100	10 (7AF &3AM)	5 (3AF &2AM) every 2 years for first 5 years	None	None	0.038 (0.071)	0.00	92	92
**9**	100	10 (7AF &3AM)	5 (3AF &2AM) every 2 years for first 5 years	4% flood	2 in 5years (1AF &1AM)	0.031 (0.074)	0.00	88	91

(Here AF – adult female, AM – adult male, r= growth rate of population, (SD)= standard deviation, N= population size at the end of 100 years, PE= probability of Extinction and H= heterozygosity of the population at the end of 100 years)

Table S2. Results of 500 simulations of population trajectories over 100 years in VORTEX (9.93) to assess the viability of swamp buffalo populations with different scenarios of carrying capacity, poaching, catastrophe, initial population size and supplementation.

Scenario	Carrying Capacity	Initial Population	Supplementation	Frequency of Catastrophes	Frequency of Harvest	r (SD)	PE	N	H%
**1**	20	10 (6AF & 4AM)	2 (1AF &1AM) every year for first 10 years	None	None	0.019 (0.155)	0.56	13	41
**2**	20	10 (6AF & 4AM)	2 (1AF &1AM) every year for first 10 years	4 % floods	None	0.019 (0.154)	0.59	13	36
**3**	20	10 (6AF & 4AM)	2 (1AF &1AM) every year for first 10 years	4% floods + 2% Diseases outbreak	2 (1AF & 1AM) every year for 100 years	0.017 (0.201)	1	0	0
4	20	10 (6AF & 4AM)	2 (1AF &1AM) every 2 years for first 10 years	None	None	0.014 (0.154)	0.62	14	38
5	20	10 (6AF & 4AM)	2 (1AF &1AM) every 2 years for first 10 years	4% floods + 2% Diseases outbreak	None	0.010 (0.162)	0.69	13	39
6	20	10 (6AF & 4AM)	2 (1AF &1AM) every 2 years for first 10 years	4% floods + 2% Diseases outbreak	2 (1AF & 1AM) every year for 100 years	0.033 (0.194)	1	0	0
7	50	10 (6AF & 4AM)	2 (1AF &1AM) every year for first 10 years	None	None	0.026 (0.107)	0.02	39	66
8	50	10 (6AF & 4AM)	2 (1AF &1AM) every year for first 10 years	4 % floods	None	0.023 (0.111)	0.06	37	66
9	50	10 (6AF & 4AM)	2 (1AF &1AM) every year for first 10 years	4% floods + 2% Diseases outbreak	2 (1AF & 1AM) every year for 100 years	0.012 (0.167)	0.93	25	60
10	50	10 (6AF & 4AM)	2 (1AF &1AM) every 2 years for first 10 years	None	None	0.023 (0.112)	0.08	39	63
11	50	10 (6AF & 4AM)	2 (1AF &1AM) every 2 years for first 10 years	4% floods + 2% Diseases outbreak	None	0.019 (0.119)	0.16	36	61
12	50	10 (6AF & 4AM)	2 (1AF &1AM) every 2 years for first 10 years	4% floods + 2% Diseases outbreak	2 (1AF & 1AM) every year for 100 years	0.024 (0.177)	0.96	30	48
13	100	20 (10AF & 10AM)	2 (1AF &1AM) every year for first 5 years	4 % floods + 2% Diseases outbreak	None	0.029 (0.090)	0.03	79	76

14	250	20 (10AF & 10AM)	None	None	2 (1AF & 1AM) every 2 years for 100 years	0.024 (0.148)	0.87	153	68
15	250	20 (10AF & 10AM)	None	4 % floods + 2% Diseases outbreak	2 (1AF & 1AM) every 2 years for 100 years	0.040 (0.162)	0.94	127	70
16	250	20 (6AF & 4AM)	2 (1AF &1AM) every year for first 5 years	None	None	0.027 (0.087)	0.02	199	79
17	250	20 (10AF & 10AM)	2 (1AF &1AM) every year for first 5 years	4 % floods + 2% Diseases outbreak	None	0.022 (0.088)	0.05	178	76
18	250	20 (10AF & 10AM)	2 (1AF &1AM) every 2 years for first 5 years	None	2 (1AF & 1AM) every 2 years for 100 years	0.018 (0.140)	0.77	159	72
19	250	20 (10AF & 10AM)	2 (1AF &1AM) every 2 years for first 10 years	4% floods + 2% Diseases outbreak	2 (1AF & 1AM) every 2 years for 100 years	0.007 (0.127)	0.71	134	72
20	250	30 (18AF & 12AM)	2 (1AF &1AM) every 2 years for first 10 years	4% floods + 2% Diseases outbreak	2 (1AF & 1AM) every 2 years for 100 years	0.008 (0.097)	0.34	162	80
21	500	10 (6AF & 4AM)	None	None	None	0.021 (0.117)	0.39	239	61
22	500	35 (20AF & 15AM)	None	None	None	0.027 (0.066)	0	396	86
23	500	35 (20AF & 15AM)	None	4% floods + 2% Diseases outbreak	None	0.023 (0.072)	0.01	337	84
24	500	35 (20AF & 15AM)	None	None	2 (1AF & 1AM) every 2 years for 100 years	0.009 (0.094)	0.32	276	78
25	500	35 (20AF & 15AM)	5 (3AF &2AM) every 2 years for first 5 years	None	None	0.029 (0.065)	0	413	89
26	500	35 (20AF & 15AM)	5 (3AF &2AM) every 2 years for first 5 years	4% floods + 2% Diseases outbreak	None	0.025 (0.069)	0	374	87

(Here AF – adult female, AM – adult male, r= growth rate of population, (SD)= standard deviation, N= population size at the end of 100 years, PE= probability of Extinction and H= heterozygosity of the population at the end of 100 years)

Fig. S1: 95% upper and lower limit suitable habitat map

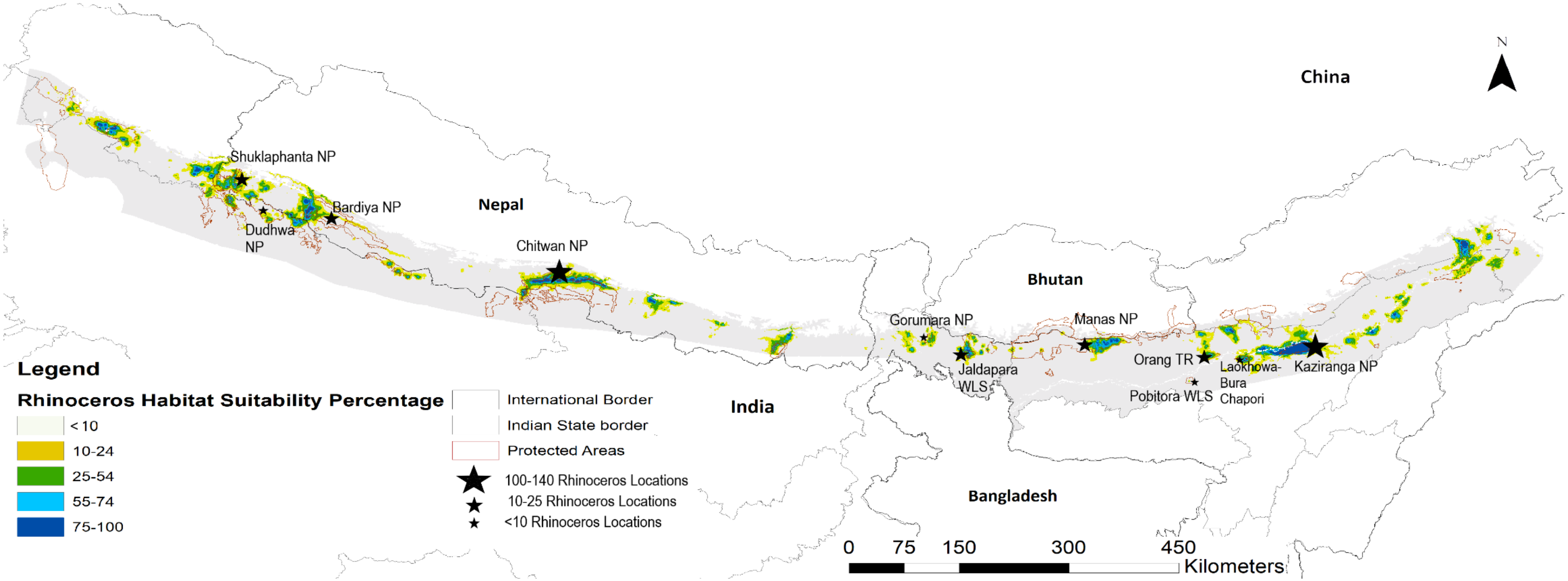

Figure a. Rhinoceros habitat suitability (Upper 95% CI) and sites sampled for occurrence locations. Created in ESRI ArcMap 10.5.1 (https://support.esri.com/en/Products/Desktop/arcgis-desktop/arcmap/10-5-1#downloads)

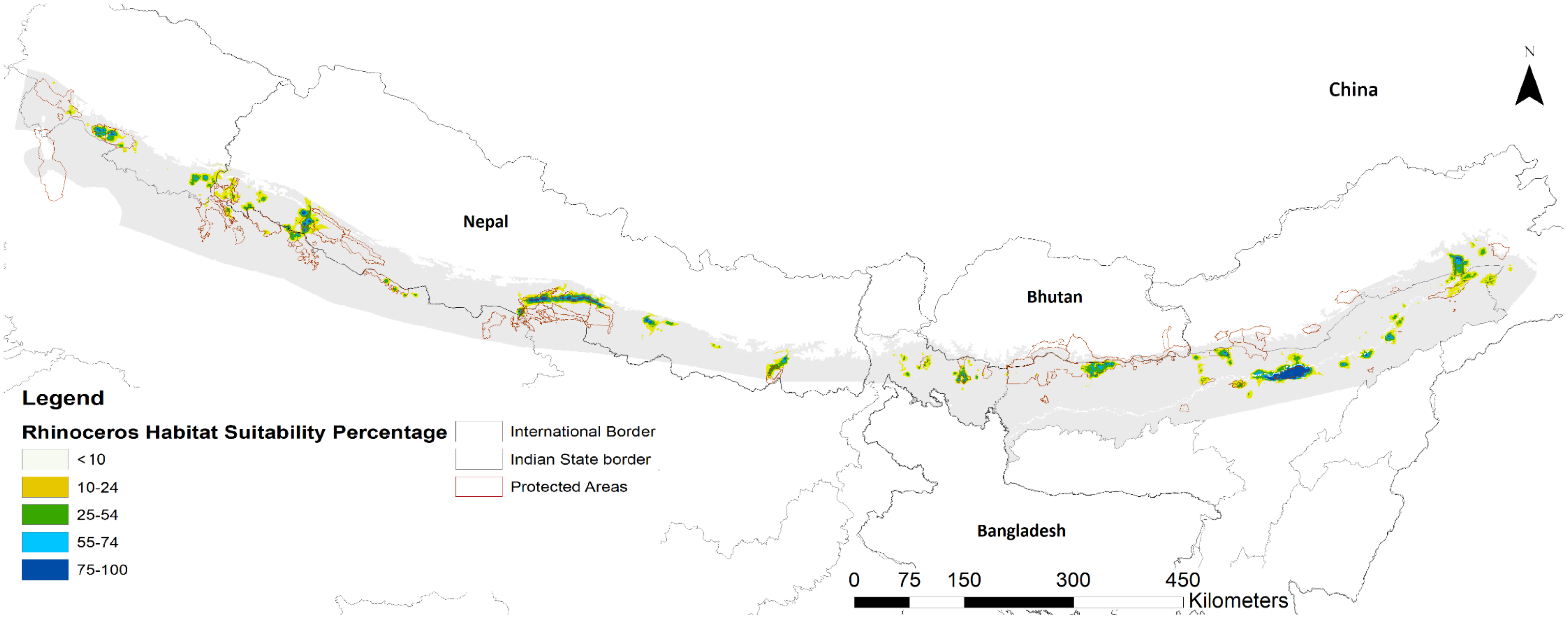

Figure b. Rhinoceros habitat suitability (Lower 95% CI). Created in ESRI ArcMap 10.5.1 (https://support.esri.com/en/Products/Desktop/arcgis-desktop/arcmap/10-5-1#downloads)

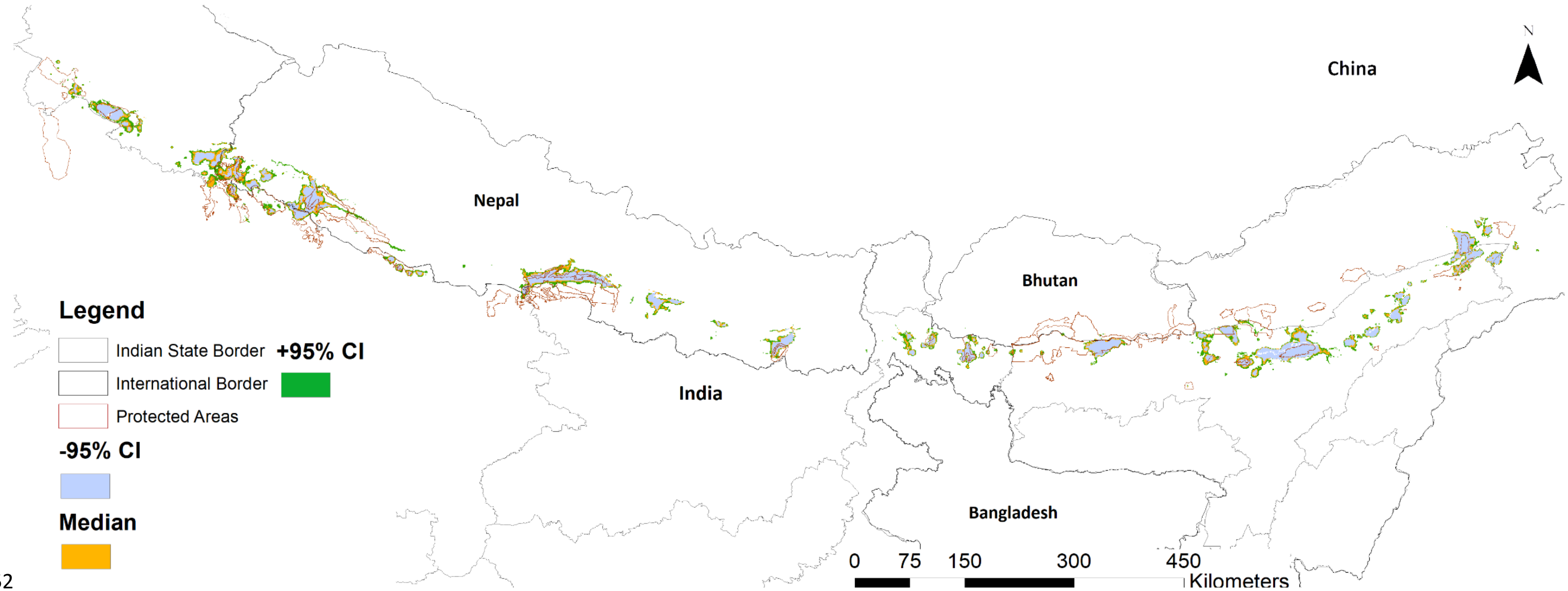

Figure c. Rhinoceros habitat suitability using Maximum training sensitivity plus specificity cumulative threshold (±95% CI). Created in ESRI ArcMap 10.5.1 (https://support.esri.com/en/Products/Desktop/arcgis-desktop/arcmap/10-5-1#downloads)

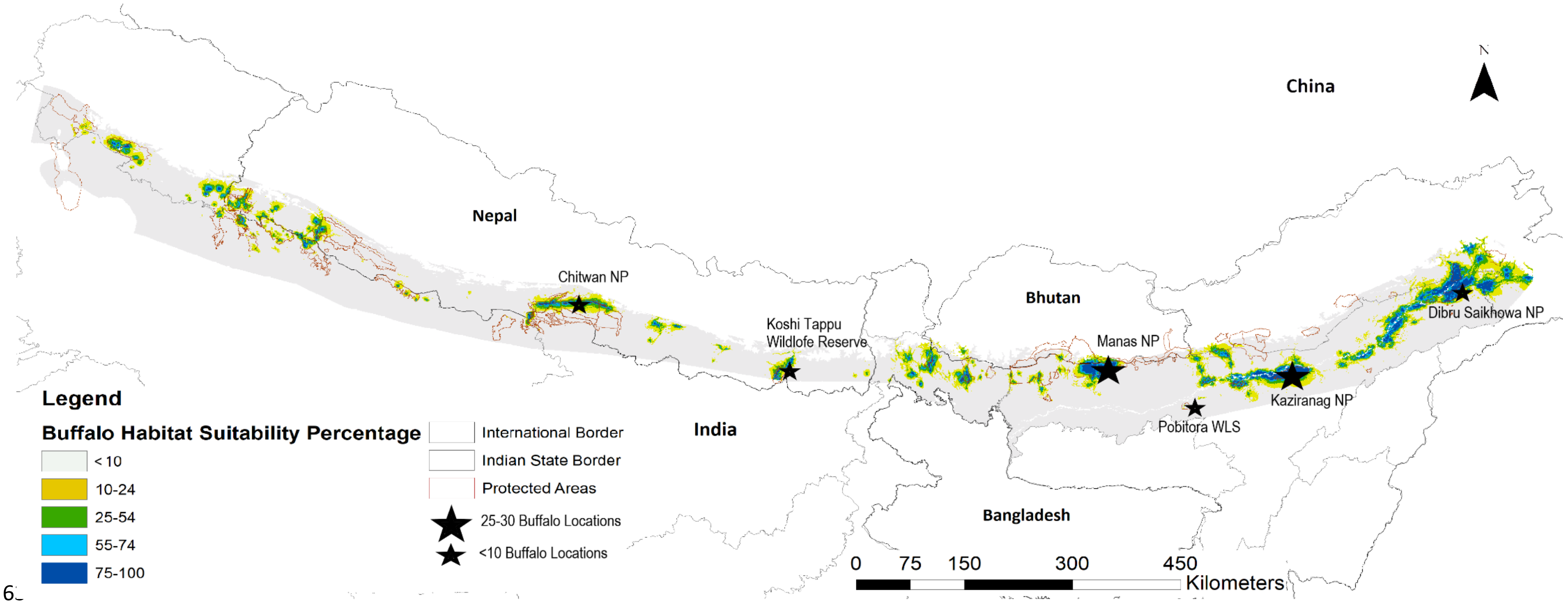

Figure d. Buffalo habitat suitability (Upper 95% CI) and sites sampled for occurrence locations. Created in ESRI ArcMap 10.5.1 (https://support.esri.com/en/Products/Desktop/arcgis-desktop/arcmap/10-5-1#downloads)

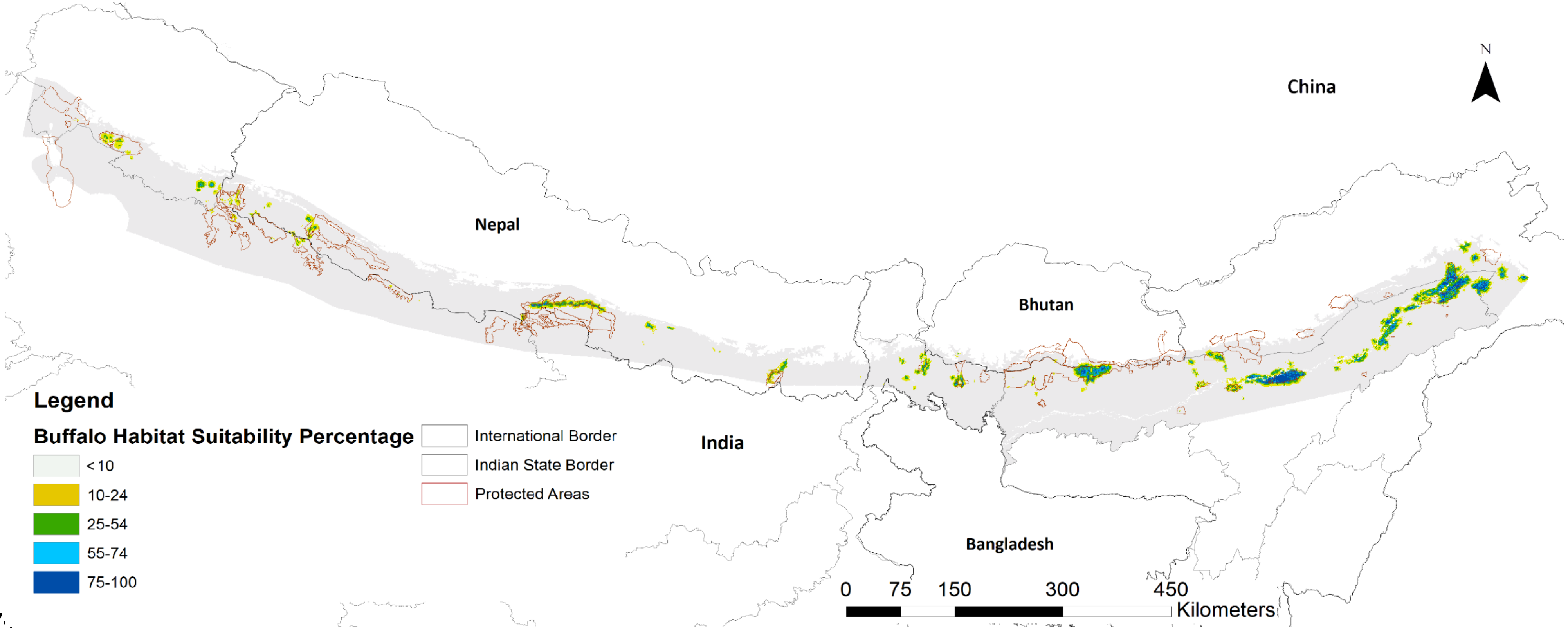

Figure e. Buffalo habitat suitability (Lower 95% CI). Created in ESRI ArcMap 10.5.1 (https://support.esri.com/en/Products/Desktop/arcgis-desktop/arcmap/10-5-1#downloads)

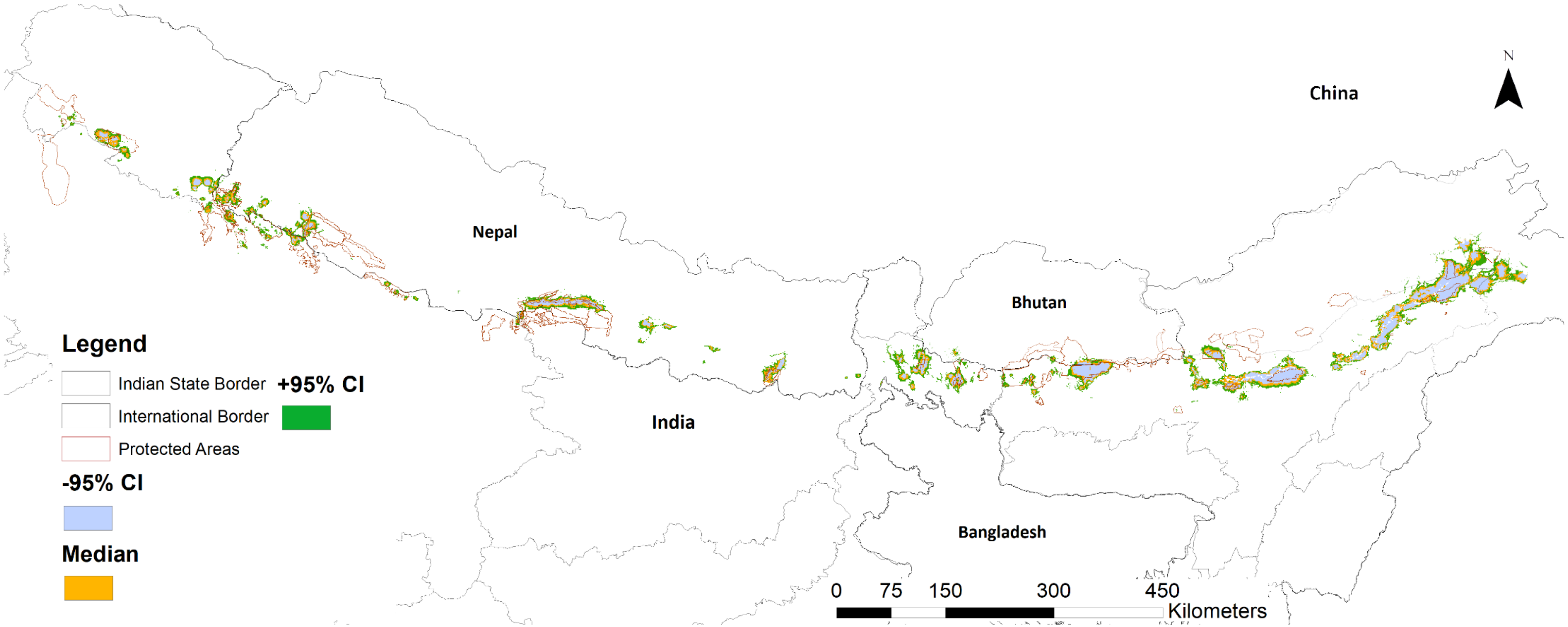

Figure f. Buffalo habitat suitability using Maximum training sensitivity plus specificity cumulative threshold (±95% CI). Created in ESRI ArcMap 10.5.1 (https://support.esri.com/en/Products/Desktop/arcgis-desktop/arcmap/10-5-1#downloads)

Table S3: Protected areas with suitable habitat for the greater one horned rhino the population they can sustain and Recommendations for Reintroduction.

Site Name	Country/ State	Protected Status	Potential Rhino Population	Positives	Negatives	Recommendation for Reintroduction
Banke	Nepal	National Park	0	-	-Human Wildlife Conflict in Buffer zone<br>- Water availability in buffer zone is low [1].	**No**, Area not found suitable along with high possibility of conflict.
Bardia	Nepal	National Park	~300	-The Karnali plains and Babai Valley can hold populations managed as metapopulation with Chitwan.<br>-Bardia can form a natural metapopulation with Shukalaphanta and Dudhwa Tiger Reserve [2]	Law enforcement, Poaching. Human-Rhino Conflict [2]	**Yes**, while strengthening protection
Laukhowa-Burachapori Complex	Assam, India	Wildlife Sanctuary complex	~90-100	-Can be maintained as a natural meta-1population with Kaziranga NP, Orang TR and Laukhowa WLS.<br>-There is Presence of Grassland species such as Swamp Buffalo -Bengal florican recorded in the past 5 years.<br>- Management of the PA is good but can be improved upon.<br>- Proposed as a potential reintroduction site by Rhino Vision 2020<br>(Personal Obs)	-High anthropogenic pressures like livestock grazing and presence of feral dogs.<br>-Agriculture around the WLS<br>-Degraded habitat with overgrazed grasslands and infested with weeds such as *Leea crispa* and *Mikenia spp* (Personal Obs)	**Yes**, but after reducing livestock pressure and improving law enforcement
Buxa	West Bengal, India	Tiger Reserve	10	-Dynamic ecosystem with constant siltation because of number of rivers that flow through.<br>- presence of other megaherbivore such as the elephant[3]	-High biotic pressures due to the presence of settlements close to the core area.	**No**, too small an area for meaningful investment required.

					-Poor protection due to inadequate staff amenities like arms, ammunition and communication equipment [3]	
Chitwan	Nepal	National Park	~850	-Currently holds a population of >600 rhinos [2]<br>- ~20 buffalos also reintroduced recently		Source Population in Nepal
Corbett	Uttrakhand, India	National Park, Tige Reserve	~250	- Effective management and protection available<br>- Good grassland habitat with Riverine forest mosaic (Personal Obs)<br>- Park is a tourist attraction, this could be used for publicising rhino conservation[3].	-High biotic pressure in the buffer area and around the PA [3].<br>- Weed infested habitats and require management[3].<br>- Local communities are not well versed with a mega herbivore like the rhino, although historically distributed in and around Corbett, the mega-grazer has been locally extinct for centuries now.	**Yes**. Can be done with minimal investments within the core area of CTR. Highly recommended for a detailed study on feasibility and planning reintroduction implementation.
Dudhwa	Uttar Predesh, India	Tiger Reserve	~70	-Successful reintroduction of Rhinos has already been done in the reserve. Moreover, the park has populations of other grassland species such as the Bengal florican and the Hispid Hare [3]<br>-Connected with similar habitats across the Indo-Nepal border, natural gene flow between populations can be maintained	- There is an ongoing problem of human-wildlife conflict due to crop raiding by ungulate species including rhinos<br>-Since the international border with Nepal is porous, increased protective framework is required [3].	Reintroduced Population needs supplementation urgently.
Gorumara	West Bengal, India	National Park	~50	- Management has undertaken programmes for habitat management, Species like sambar, spotted deer and gharial have been introduced and monitored closely [3].<br>-Patrolling is also carried out regularly by vehicle and on elephant back. Thus, suggesting effective management system<br>- The staff are well trained in management and Conservation<br>-Population of ~50 rhinos exist in the park [4,5]	-Large number of vacancies in staff that are filled with casual workers<br>- Anthropogenic pressures around the park, may lead to human-wildlife conflict [3]	Current population is close to 50; Predicted K =57. However, conservation investments can lead to higher densities as seen in the case of Kaziranga.

Jaldapara	West Bengal, India	Wildlife Sanctuary	~230	-Rhino population along with population of several other ungulates such as gaur and sambar have been on the increase[6].<br>- Currently the Rhino population is estimated to be 237 [5], making Jaldapara the second largest rhino population in the country[6].	- The habitat is prone to high levels of biotic pressures from surrounding settlements.<br>- Number of cases of human-wildlife conflict have already been reported from the surrounding areas [6].	Current population is close to 237 (at predicted K), suggesting that the population is healthy.
Katerniaghat	Uttar Predesh, India	Wildlife Sanctuary	~130	Currently rhinos from Bardia occasionally cross over [6].	Law Enforcement and high potential of Human-Rhino conflict [6].	**Yes**, with electric fencing of sensitive crop field - PA interface
Kaziranga	Assam, India	National Park	~2400	- Good habitat with grassland forest mosaic and swamps. Furthermore, the size of the NP can hold a sizable population of rhinos.<br>- Other ungulate densities are also high indicating the grassland have high productivity and the park has good protection (75 vehicles and 25 boats for patrolling<br>- Human-Rhino conflict is negligible<br>-Current mortality due to humans less than replacement, thus population trend is increasing (Kaziranga Field Director 2018, Personal communication).	-Weed infestation has degraded that habitat. Infestation of water hyacinth has blocked natural water channels [3]<br>- Biotic Pressures around the Park are causing rapid habitat degradation in the surrounding areas)<br>- National Highway 37 is a major threat to wildlife crossing into the highlands [3]; Kaziranga Field Director 2018, Personal communication).	Largest Source population in India (Population ~ predicted K, healthy population)

Kishanpur	Uttar Pradesh, India	Wildlife Sanctuary	1	- It forms a continuous tract of forest as it is connecter to Pilibhit tiger reserve.<br>- The Park is blessed with mosaic habitat with grasslands and forests of sal and teak. It also has a healthy population of swamp deer, which can be used as a surrogate for rhino habitat [6].	-Although, the park has a number of human settlements in and around the boundaries.<br>- Furthermore, there is a highway that cuts across the central region of the park and there is a railway line passing through the park as well. Both these serve as barrier to wildlife and have been the cause of several animal mortalities [7].	**No**, Too small an area for meaningful investments.
Koshi Tappu	Nepal	Wildlife Reserve, RAMSAR Site	~75	- Thriving population of Swamp Buffalos [8].	- High biotic pressure in and around the park [8].	**Yes.** But not a priority requires i) investing in law enforcement<br>ii) Human-Rhino mitigation measures
Manas	Assam, India	National Park	~530	- Vast grassland habitats available<br>- Populations of megaherbivores such as buffaloes, Gaur and elephants also present.<br>- Management well versed with reintroduction protocol and has anti-poaching camps set up as rhino reintroduction was undertaken in 2007 (Barman et al. 2014; Personal Obs)	- Grasslands are rapidly being infested by weeds such as *Leea cirspa* and *Leea indica.*<br>- As the rivers in Manas are not as dynamic as the Brahmaputra, there is no annual siltation, therefore the nutrient quality of the grasslands is constantly decreasing<br>- Current management is not very keen on bringing more rhinos into the park	Current Rhino population of 30 individuals, established by reintroduction (40 individuals) after their local extinction due to poaching during the civil unrest.<br>**Yes**, High potential for a large population. Needs better enforcement mechanism and some

					- Of 40 translocated individuals in 2007, the population today is around 30 animals.<br>- There is a constant threat of a civil unrest in the region also with predominant poaching of animals<br>- Although the park enjoys a huge buffer zone, the administrative rights over this area are split between the forest divisions of Kanchugaon, Haltugaon, Chiran, Buksa and Dhans, thus making administration of the area very difficult [3].	power fencing of sensitive boarders with PA and agriculture/villages
Orang	Assam, India	Tiger Reserve	~50	- Can be maintained as a meta-population with Kaziranga NP, Laukhowa WLS and Burachapori WLS (Personal Obs).<br>- Grassland habitat has little weed infestation (L*eea crispa)* and has the potential to support higher rhino population than current one<br>- Management and protection level seem good<br>(Personal Obs)	- Surrounded by human dominated landscape thus increasing the chances of human-rhino conflict.<br>- The park has low densities of ungulates, however has highest recorded tiger densities in the world ([7]; Personal Obs).	Current population of close to 80 individuals. Can benefit by artificial supplementation of a few 5-10 rhinos from Kaziranga.<br>Power fencing of sensitive boarders with agriculture and PA are required.<br>Park has potential to sustain higher densities.
Parsa	Nepal	Wildlife Sanctuary	~30	-Connectivity to Chitwan National Park.<br>-2015 survey reported first rhino presence (3 individuals) [2].	-High biotic pressures [2].	**Yes**. Can be maintained as a metapopulation with Chitwan and Valmiki
Pilibhit	Uttar Predesh, India	Tiger Reserve	~120	-Acts as a natural corridor between Shukalaphanta in Nepal and Kishanpur WLS in India.<br>-Provides connectivity to Lagga bagga forest [3].<br>- The 2014 tiger survey suggests Pilibhit as a prominent tiger habitat (Jhala et al. 2014).	- However, since the pa is used as a corridor, human wildlife conflict remains high.<br>-The park also has high levels of biotic and anthropogenic pressures [3].	**Yes**. After conflict mitigation measures are in place since linear nature of the reserve would cause severe conflicts with neighbouring human habitation and agriculture. Can be maintained as

						metapopulation with Dudhwa, Shukalaphanta, Bardia and Katerniagath.
Pobitora	Assam, India	Wildlife Sanctuary	*~100	-Current Rhino population is close to 100 animals<br>-High density of Swamp Buffalo also present (Personal Obs).	-High biotic pressure in and around the park<br>-Heavy cattle grazing inside the park.<br>- Instances of Crop-raiding by rhinos and buffalos reported frequently (Personal Obs)	- Despite the small area (38.8 Km^2), the park harbours high density of both rhinos and buffalos.<br>-Law enforcement required.
Rajaji	Uttarakhand, India	National Park	~70	- The park is forming a crucial part of the landscape, providing connectivity from Corbett National Park, westwards end of the Terai landscape [3].	-The protected area suffers immensely dude to the presence of local cattle herders (Gujars) and their livestock.<br>- The park has several state and district roads cutting across and is surrounded by large settlements, cities and townships [3].	**No**. Isolate population close to human habitations on River Ganges and its tributaries, High potential for conflict.
Royal Manas	Bhutan	National Park	~15	- Although suitable habitat is available it is small in size. Contiguous rhino habitat exists between Royal Manas and Indian Manas (Personal Obs).	- The management is deficient in manpower and thus is not keen on rhino reintroduction (Personal Obs).	**Not in the near future.** Though it may be of interest as a National Pride.
Sohagibarwa	Uttar Pradesh, India	Wildlife Sanctuary	<10	- The protected area has natural connectivity to Valmiki and Chitwan, Nepal [3].	- In 2013 survey it was observed that the protected area has around 300 villages inside and around its boundaries, thus accounting to high biotic pressures.<br>-There is also the problem of lack of funding [3].	**No**. Too small an area.
Sohelwa	Uttar Pradesh, India	Wildlife Sanctuary	~100	- Park is of great conservation value as it harbours a number of threatened species [6].	- Extensive biotic pressures.<br>- there is also the problem of human wildlife conflict that is ever increasing [6].	**Not in the near future**. Investments required are large. But if the PA manages to address its current problems, Rhino introduction can be considered in the future.
Sonai Rupai	Assam, India	Wildlife Sanctuary	~130	-Good habitat available<br>- Investments made to improve protection and reduce biotic pressure [6].	-There is a lack of alternative livelihoods and husbandry practices and local communities still depend on forest resources [6].	**Yes**, However, there is a need to provide alternate livelihoods and husbandry

						practices to the local communities.
Shukalaphanta	Nepal	National Park	~270	- 2015 survey reported 8 Rhinos [2]	- Problem of invasive species, Protection [2].	Current Population of 8 rhinos exists. Substantial population was poached during civil unrest. More rhinos need to be supplemented from Chitwan
Valmiki	Bihar, India	Tiger Reserve	~40	- Natural connectivity to Chitwan National Park in Nepal.<br>-Management has good leadership [3].	- High Biotic pressures.<br>- A 6km long railway line operates inside the park. However, the management has proposed to the state government of Bihar to relocate the railway line outside the protected area.<br>- Protection is poor as there is over 80% vacancy for staff, furthermore no armed guards present currently, thus increasing the chances of poaching [3].	**Yes**. But after appropriate mitigation by relocating the railway line, increased infrastructure for law enforcement.
Dibru Saikhowa	Assam, India	National Park	~160	-Extant buffalo population<br>-Good productive habitat<br>-Identified by Rhino vision 2020 as potential site for reintroduction [10].	- Reduction in anthropogenic pressure is required<br>- Increased in level of protection	**Yes,** after anthropogenic pressures are reduced and protection increased.

Fig S2: Individual response curves for a) Rhinoceros b) Buffalo

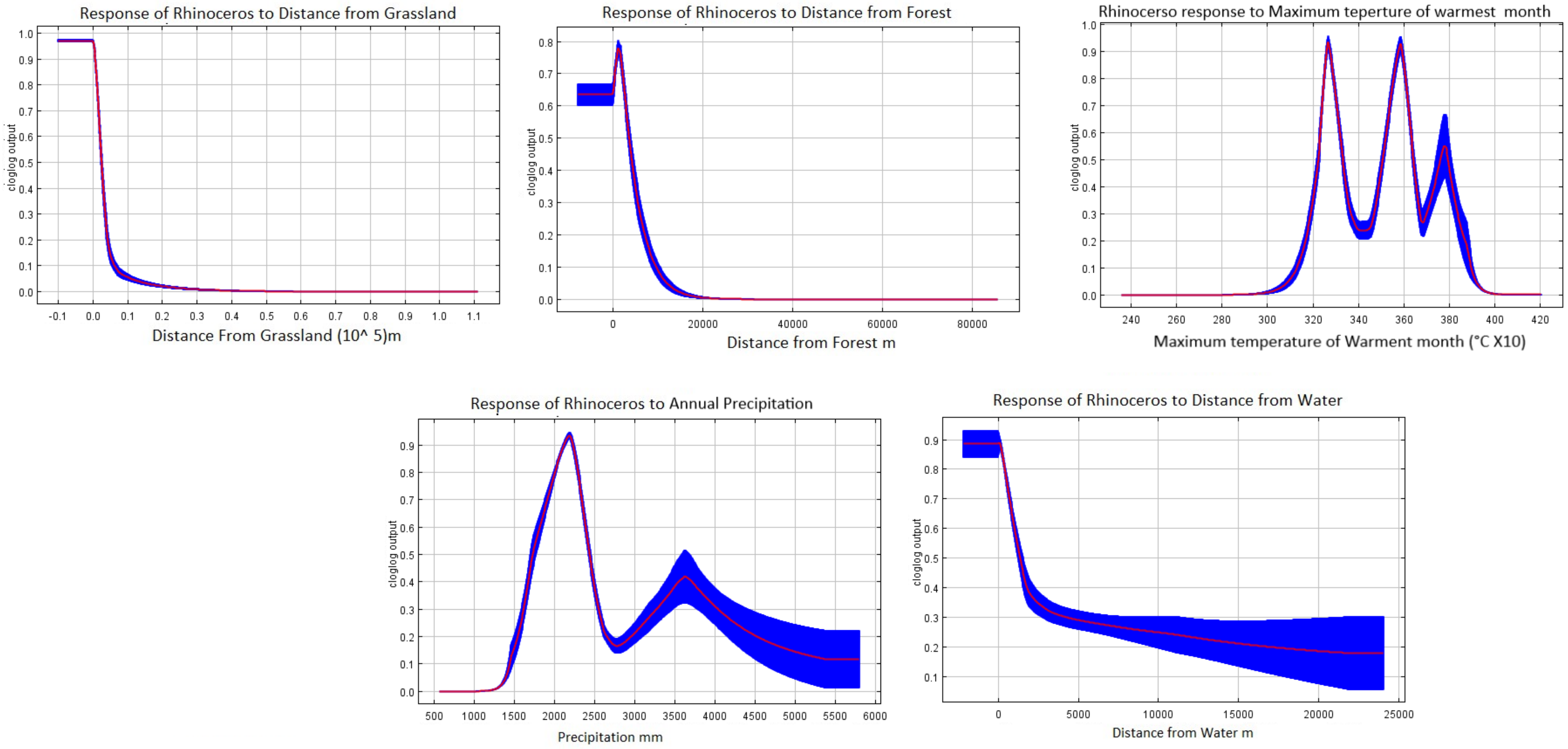

Figure a. Rhinoceros Individual response curves

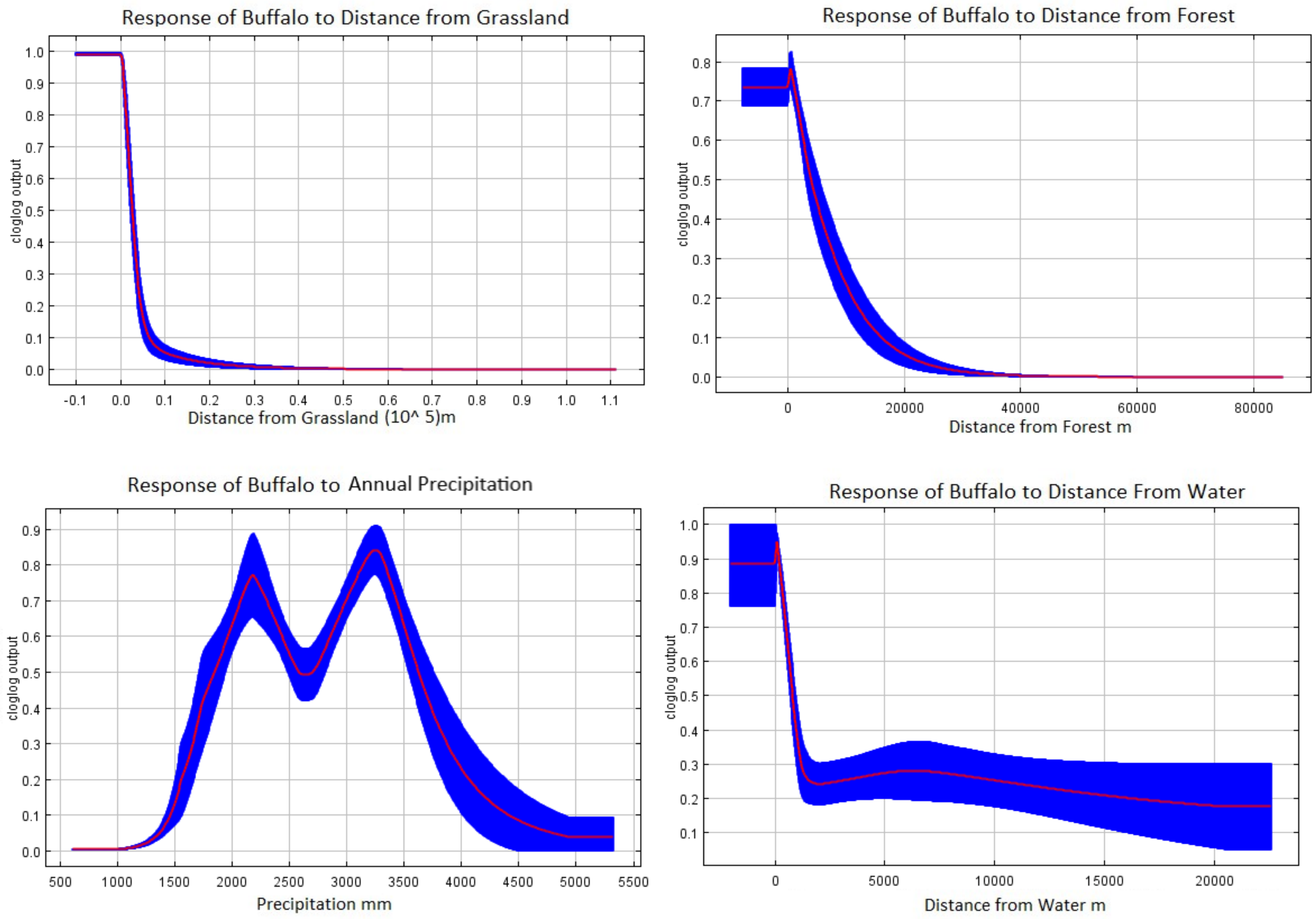

Figure b. Buffalo individual response curves

Fig S3: Picture from Laokhowa Wildlife Sanctuary of livestock grazing in grasslands inside the protected area. The picture shows heavily weed infested grassland which is overgraze

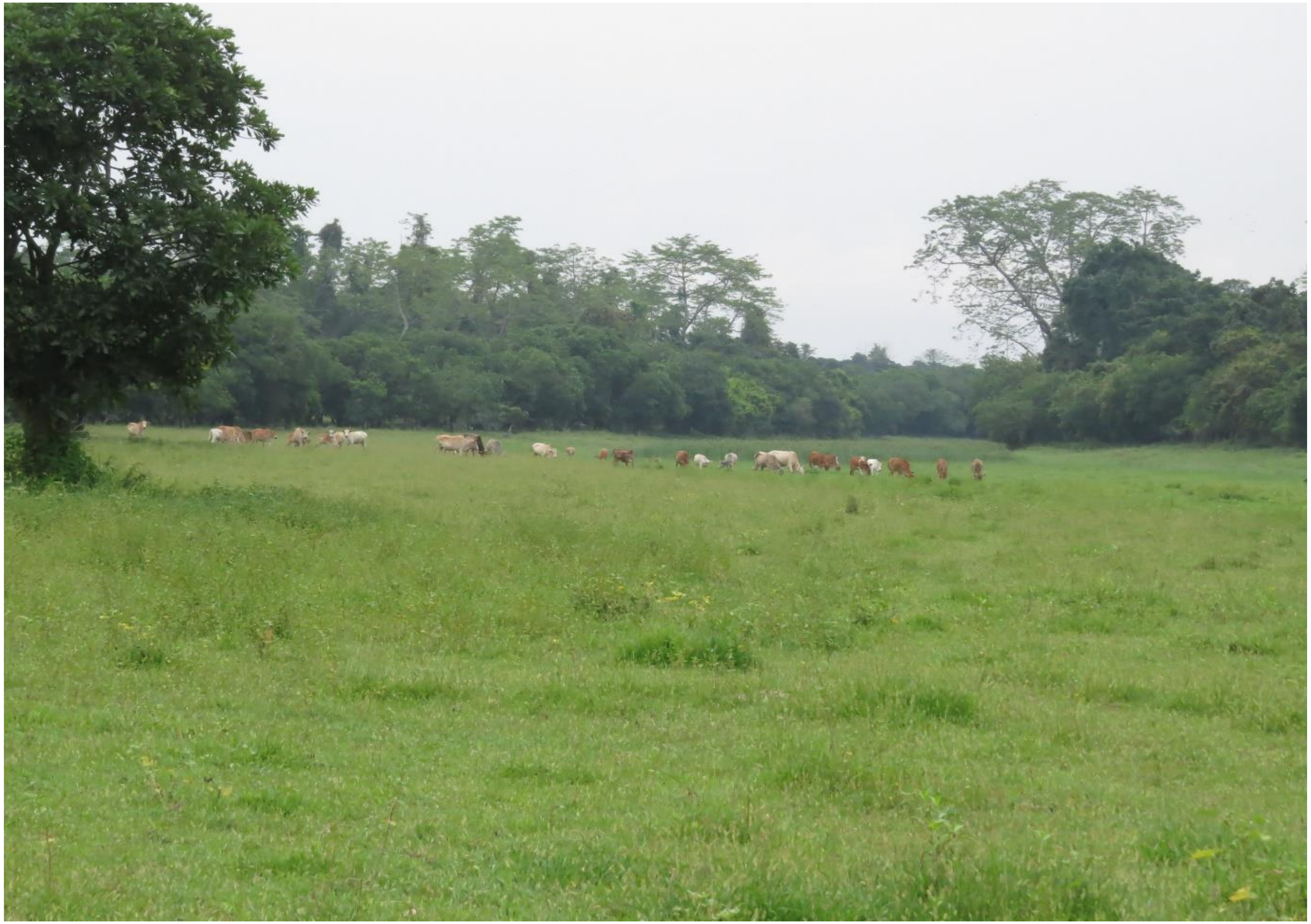

Fig S4: Picture of grassland from Pobitora Wildlife Sanctuary showing Megaherbivores (Rhinos and Buffalos) grazing alongside domestic livestock. This is due to the small size of the reserve and high human densities in surrounding area.

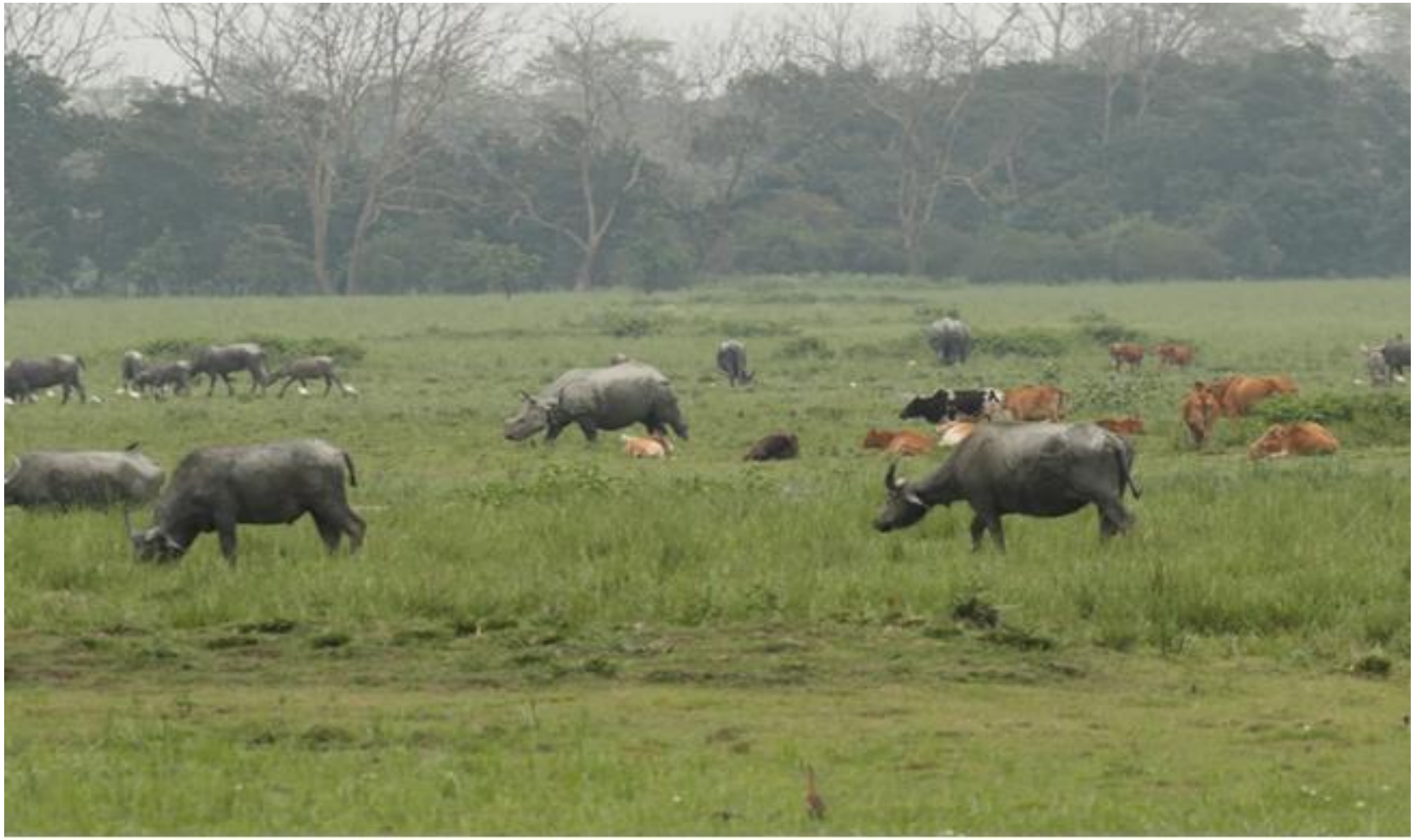

Fig S5: Buffalo to Maximum Temperature of Warmest month

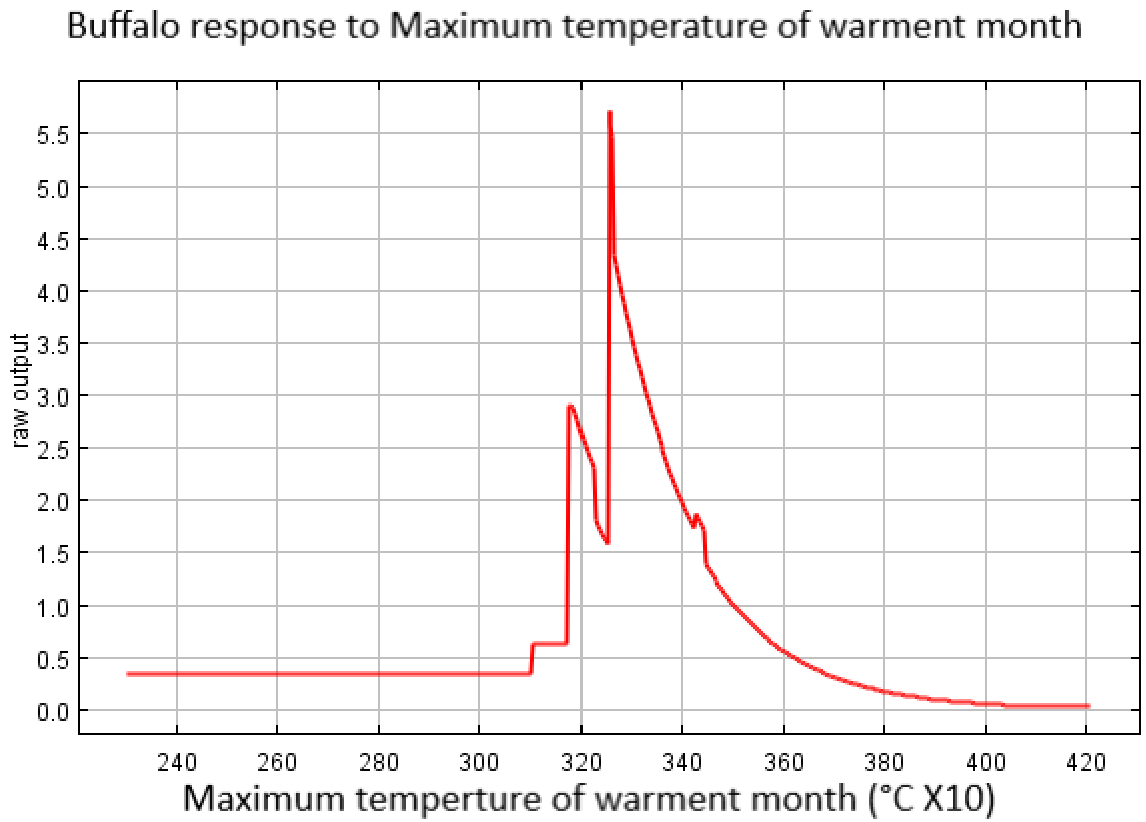

Fig S6: Maps of Grassland polygons with ground validation locations (accuracy of 88.6%)

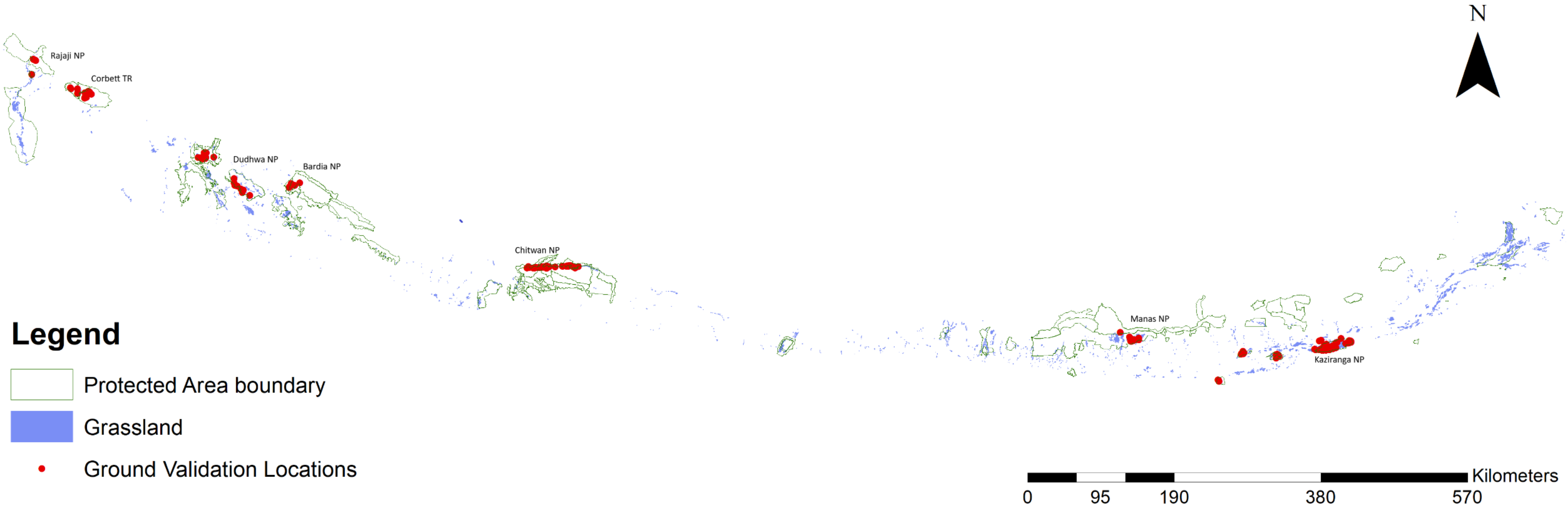

Figure S 6a. Grassland polygons with ground validation locations (with 88.6% accuracy). Created in ESRI ArcMap 10.5.1 (https://support.esri.com/en/Products/Desktop/arcgis-desktop/arcmap/10-5-1#downloads)

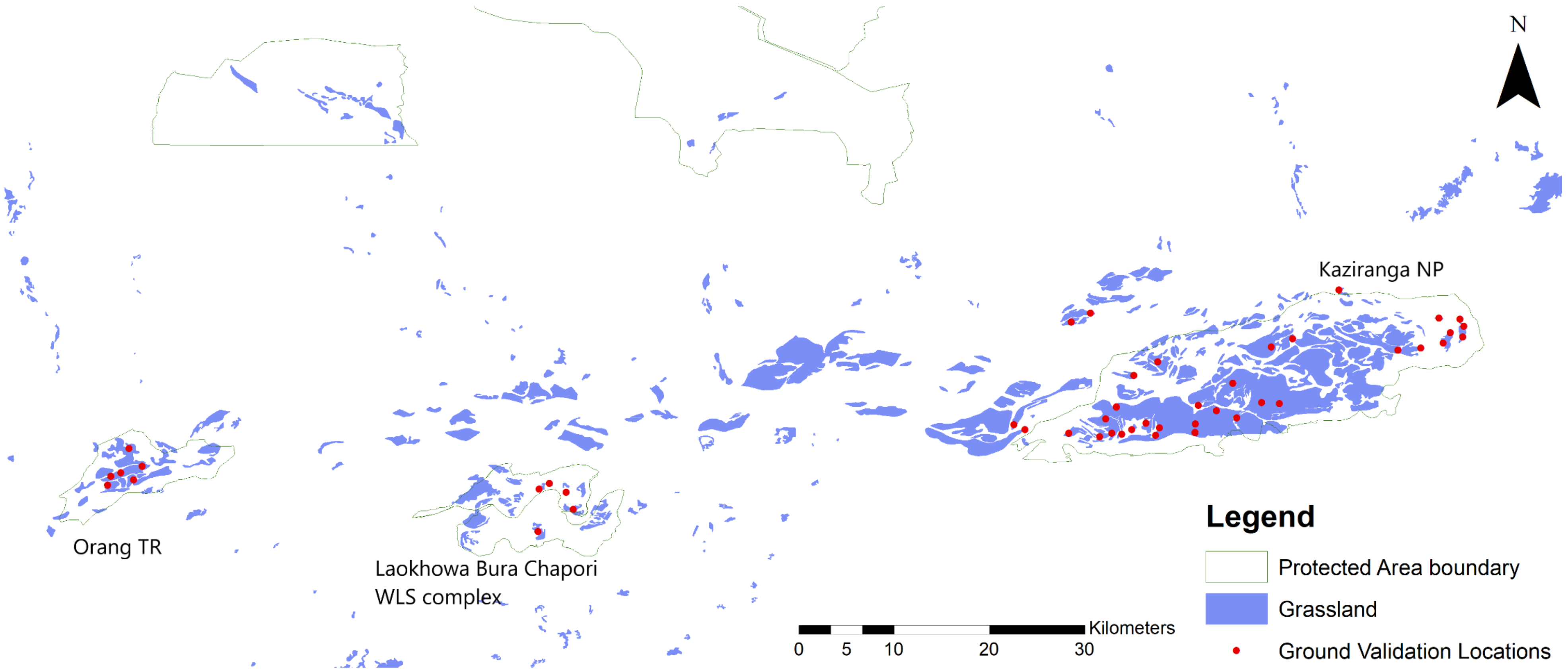

Figure S 6b. Grassland polygons with ground validation locations (zoomed to Kaziranga NP, Orang TR and Laokhowa Bura Chapori WLS complex). Created in ESRI ArcMap 10.5.1 (https://support.esri.com/en/Products/Desktop/arcgis-desktop/arcmap/10-5-1#downloads)

Table S4: Correlation matrix between variables/covariates used in species distribution modelling in MaxEnt.

	AP	PW	PQ	MXT	MIT	E	DF	DGL	HF	DNDVI	DPA	PONDVI	PRNDVI	DW
**AP**	1.00	0.88	0.26	-0.79	0.43	-0.05	-0.45	-0.05	-0.09	-0.14	-0.16	0.34	0.55	-0.20
**PW**	0.88	1.00	0.04	-0.54	0.26	0.04	-0.37	-0.11	-0.10	-0.05	-0.17	0.27	0.39	-0.07
**PQ**	0.26	0.04	1.00	-0.19	-0.21	0.23	-0.36	-0.15	-0.05	0.01	-0.23	0.24	0.30	-0.02
**MXT**	-0.79	-0.54	-0.19	1.00	-0.37	-0.16	0.53	-0.11	0.18	-0.04	0.00	-0.48	-0.58	0.16
**MIT**	0.43	0.26	-0.21	-0.37	1.00	-0.73	-0.03	0.07	0.24	-0.34	0.07	-0.14	0.11	-0.36
**E**	-0.05	0.04	0.23	-0.16	-0.73	1.00	-0.33	0.04	-0.38	0.44	-0.08	0.46	0.21	0.28
**DF**	-0.45	-0.37	-0.36	0.53	-0.03	-0.33	1.00	0.12	0.28	-0.31	0.27	-0.51	-0.39	-0.05
**DGL**	-0.05	-0.11	-0.15	-0.11	0.07	0.04	0.12	1.00	-0.05	-0.04	0.45	0.03	0.06	-0.01
**HF**	-0.09	-0.10	-0.05	0.18	0.24	-0.38	0.28	-0.05	1.00	-0.30	0.07	-0.39	-0.24	-0.18
**DNDVI**	-0.14	-0.05	0.01	-0.04	-0.34	0.44	-0.31	-0.04	-0.30	1.00	-0.08	0.62	-0.06	0.30
**DPA**	-0.16	-0.17	-0.23	0.00	0.07	-0.08	0.27	0.45	0.07	-0.08	1.00	-0.14	-0.11	-0.05
**PONDVI**	0.34	0.27	0.24	-0.48	-0.14	0.46	-0.51	0.03	-0.39	0.62	-0.14	1.00	0.75	0.24
**PRNDVI**	0.55	0.39	0.30	-0.58	0.11	0.21	-0.39	0.06	-0.24	-0.06	-0.11	0.75	1.00	0.04
**DW**	-0.20	-0.07	-0.02	0.16	-0.36	0.28	-0.05	-0.01	-0.18	0.30	-0.05	0.24	0.04	1.00

Here, AP= Annual Precipitation MXT= Maximum temperature of hottest month, MIT= Minimum temperature of coldest month, PW= Precipitation of wettest month, PQ= Precipitation of driest quarter, E= Elevation, DW= Distance from Water, DF= Distance from forest, DGL= Distance from grassland, HF= Human Footprint, DPA= Distance from Protected area, PONDVI= Post monsoon NDVI, PRNDVI= Pre-monsoon NDVI and DNDVI= Difference in Post and Pre-monsoon NDVI.

Table S5: Parameters for PHVA and Sources

a) Greater One-horned Rhinoceros

Population and habitat parameters	Values Used	References/Sources
Age of first offspring of female (year)	8	[11]
Age of first offspring of Male (year)	10	[11]
Maximum age of reproduction	30	[12]
Percentage of females breeding each year	33	[11–13].
% available for breeding at K	80	[14]
% available for breeding below carrying capacity	100	[14]
Sex ratio at birth (%)	50	[11]
Mortality in 0-1 years ± SD (both sex)	23 ± 2.4 (%)	[14]
Female Survival for 1-6 years ± SD	0.962 ± 0.012	[14]
Female Survival for 6-8 years ± SD	0.98 ± 0.008	[14]
Female Survival after 8 years ± SD	0.985 ± 0.008	[14]
Male Survival for 1-6 years ± SD	0.965 ± 0.01	[14]
Male Survival for 6-8 years ± SD	0.985 ± 0.008	[14]
Male Survival for 8-10 years ± SD	0.985 ± 0.008	[14]
Male Survival after 10 years ± SD	0.984 ± 0.001	[14]
% Males in the breeding pool	30	Assumption based on field Observations

b) Swamp Buffalo

Population and habitat parameters	Values Used	References/Sources
Age of first offspring of female (year)	3	[8,15]
Age of first offspring of Male (year)	3	[8,15]
Maximum age of reproduction	23	[8,15]
Percentage of females breeding each year	50	[8,15]
% available for breeding at K	80	Assumption
% available for breeding below carrying capacity	100	Assumption
Sex ratio at birth (%)	50	[8,15]
Mortality in 0-1 years ± SD (both sex)	30 ± 5 (%)	[16]
Female Survival for 1-2 years ± SD	0.90 ±0.02	[16]
Female Survival for 2-3 years ± SD	0.90 ± 0.02*	[16]
Female Survival after 3 years ± SD	0.90 ± 0.02	[16]
Male Survival for 1-2 years ± SD	0.86 ± 0.03	[16]
Male Survival for 2-3 years ± SD	0.78 ± 0.03	[16]
Male Survival after 3 years ± SD	0.89 ± 0.03	[16]
% Males in the breeding pool	40	Assumption

References:

1. Ayadi, D. P. Human-wildlife conflict in buffer zone area: a study of Banke National Park, Nepal. 63 (2011).
2. *DNPWC.Rhino Count Report 2015*. (2015).
3. Mathur, V., Gopal, R., Yadav, S. & Sniha, P. *Management Effectiveness Evaluation (MEE) of Tiger Reserves in India: Process and Outcomes*. (2011).
4. Mukherjee, T., Sharma, L. K., Saha, G. K., Thakur, M. & Chandra, K. Past, Present and Future: Combining habitat suitability and future landcover simulation for long-term conservation management of Indian rhino. *Sci. Rep.* (2020). doi:10.1038/s41598-020-57547-0
5. Rhinoceros, I. Estimation of Indian Rhinoceros.
6. Mathur, V. ., Gopal, R., Yadav, S. ., Negi., H. . & Ansari, N. . *Management Effectiveness Evaluation (MEE) of Tiger Reserves in India*. (2015).
7. Jhala, Y., Qureshi, Q. & Gopal, R. *STATUS OF TIGERS, COPREDATORS & PREY IN INDIA, 2014*. (2015).
8. Khatri, T. B., Shah, D. N. & Mishra, N. Wild Water Buffalo Bubalus arnee in Koshi Tappu Wildlife Reserve , Nepal : status , population and conservation importance. *J. Threat. Taxa* **4**, 3294–3301 (2012).
9. Barman, R., Choudhury, B., Ashraf, N. V. K. & Menon, V. Rehabilitation of greater one-horned rhinoceros calves in manas national park, a world heritage site in India. *Pachyderm* **55**, 79–88 (2014).
10. Singh, S. P., Sharma, A. & Talukdar, B. K. Translocation of Rhinos within Assam : A successful third round of the second phase of translocations under Indian Rhino Vision (IRV) 2020. 1–6 (2012).
11. Dinerstein, E. & Price, L. Demography and Habitat Use by Greater One-Horned Rhinoceros in Nepal. *J. Wildl. Manage.* **55**, 401 (1991).
12. Subedi, N. Effect of Mikania micrantha on Demography, Habitat use and Nurtition of Greater one-horned rhinocerosceros in Chitwan National Park. (Forest Research Institute, 2012).
13. Laurie, A. the Ecology and Behaviour of the Greater One-Horned Rhinoceros. *Behaviour* (1978).
14. Subedi, N., Lamichhane, B. R., Amin, R., Jnawali, S. R. & Jhala, Y. V. Demography and viability of the largest population of greater one-horned rhinoceros in Nepal. *Glob. Ecol. Conserv.* **12**, 241–252 (2017).
15. Hedges, S., Sagar Baral, H., Timmins, R. . & Duckworth, J. . Bubalus arnee. *IUCNRed List Threat. Species 2008* (2008).
16. Heinen, J. T. & Kandel, R. Threats to a small population: A census and conservation recommendations for wild buffalo Bubalus arnee in Nepal. *Oryx* **40**, 324–330 (2006).
